# Nitric oxide has contrasting age-dependent effects on the functionality of murine hematopoietic stem cells

**DOI:** 10.1186/s13287-016-0433-x

**Published:** 2016-11-22

**Authors:** Sapana Jalnapurkar, Shweta Singh, Moirangthem Ranjita Devi, Lalita Limaye, Vaijayanti Kale

**Affiliations:** Stem Cell Lab, National Centre for Cell Science, Ganeshkhind, Pune, 411007 India

**Keywords:** Nitric oxide, CD34, Hematopoietic stem cells, Engraftment, Age-dependent, Self-renewal, Commitment, Transcription factors

## Abstract

**Background:**

The success of hematopoietic stem cell (HSC) transplantation is dependent on the quality of the donor HSCs. Some sources of HSCs display reduced engraftment efficiency either because of inadequate number (e.g., fetal liver and cord blood), or age-related dysfunction (e.g. in older individuals). Therefore, use of pharmacological compounds to improve functionality of HSCs is a forefront research area in hematology.

**Methods:**

Lineage negative (Lin^−^) cells isolated from murine bone marrow or sort-purified Lin^−^Sca-1^+^c-Kit^+^CD34^−^ (LSK-CD34^−^) were treated with a nitric oxide donor, sodium nitroprusside (SNP). The cells were subjected to various phenotypic and functional assays.

**Results:**

We found that SNP treatment of Lin^−^ cells leads to an increase in the numbers of LSK-CD34^+^ cells in them. Using sort-purified LSK CD34^−^ HSCs, we show that this is related to acquisition of CD34 expression by LSK-CD34^−^ cells, rather than proliferation of LSK-CD34^+^ cells. Most importantly, this upregulated expression of CD34 had age-dependent contrasting effects on HSC functionality. Increased CD34 expression significantly improved the engraftment of juvenile HSCs (6–8 weeks); in sharp contrast, it reduced the engraftment of adult HSCs (10–12 weeks). The molecular mechanism behind this phenomenon involved nitric oxide (NO)-mediated differential induction of various transcription factors involved in commitment with regard to self-renewal in adult and juvenile HSCs, respectively. Preliminary experiments performed on cord blood-derived and mobilized peripheral blood-derived cells revealed that NO exerts age-dependent contrasting effects on human HSCs as well.

**Conclusions:**

This study demonstrates novel age-dependent contrasting effects of NO on HSC functionality and suggests that HSC age may be an important parameter in screening of various compounds for their use in manipulation of HSCs.

**Electronic supplementary material:**

The online version of this article (doi:10.1186/s13287-016-0433-x) contains supplementary material, which is available to authorized users.

## Background

The success of hematopoietic stem cell (HSC) transplantation is dictated by the quality of the donor HSCs, and is dependent on their ability to home to the bone marrow (BM) of recipients, self-renew, and differentiate. Similarly, the HSCs differentiated from embryonic stem (ES) cells or induced pluripotent stem (iPS) cells fail to engraft irradiated hosts [1–3]. Hence, two principal approaches are taken to improve the engraftment of donor HSCs; one, therapeutic targeting of the HSC niche [4, 5], and two, direct treatments of HSCs [6–10].

Nitric oxide (NO), a short-lived free radical, regulates several aspects of hematopoiesis in a development-dependent manner. It has been shown to play an important role in hematopoietic development in the embryonic stage [11]. Silencing of genes encoding NO synthase (NOS) blocked HSC development which could be rescued by treatment with NO donors. NO is also known to play a critical role in shear stress-induced augmentation of hematopoiesis from mouse ES cells [12].The importance of NO in adult hematopoiesis was underscored by the finding that mice deficient in NOS3 show reduced hematopoietic recovery and impaired mobilization of endothelial cells [13, 14]. Conversely, treatment of adult mice with NOS inhibitors increased the number of stem cells, suggesting that NO limits HSC proliferation [15]. In vitro treatment of adult BM cells with NO donors inhibits erythroid progenitors while stimulating myeloid progenitors [16, 17]. However, direct effects of NO donors on HSC functionality were not examined.

Here we show that treatment of LSK-CD34^−^ HSCs with NO donors upregulates CD34 expression in them through a cGMP-independent pathway. Importantly, the upregulated expression of CD34 was associated with self-renewal and gain of functionality in the juvenile HSCs (6–8 weeks), whereas it was associated with lineage commitment and loss of functionality in the adult HSCs (10–12 weeks). These data demonstrated that NO has age-dependent contrasting effects on murine HSCs. Preliminary experiments performed on cord blood (CB) and mobilized peripheral blood leukocytes (MBPL) also showed that NO exerts age-dependent effects on human HSCs as well.

Our data suggest that NO donors could be used to improve the engraftment ability of HSCs derived from younger sources such as ES/iPS cells (embryonic), fetal liver (fetal), and cord blood (neonatal), but their use in manipulation of adult HSCs may be avoided.

## Methods

### Animals

Protocols for animal experiments were approved and were performed in accordance with the Institutional Animal Ethical Committee (IAEC) of the National Centre for Cell Science (NCCS) and the Committee for the Purpose of Control and Supervision of Experiments on Animals (CPCSEA) (approval number: EAF/2010/B-151). Throughout the experiments animals were under the supervision of trained personnel. C57BL/6 J (CD45.2) and B6.SJL- Ptprc^a^ Pep3^b^/BoyJ (CD45.1) mice purchased from Jackson Laboratory (Bar Harbor, ME, USA) were housed and bred in the institutional experimental animal facility. Mice at 6–8 or 10–12 weeks of age were used in the experiments.

### Cell culture and treatments

BM-derived mononuclear cells (MNCs) of Ptprc (CD45.1) mice were depleted of lineage-positive cells using the immuno-magnetic technique (Additional file 1: Table S1). This HSC-enriched lineage-negative fraction, or LSK-CD34^−^ cells sorted from them, were cultured in Iscove’s modified Dulbecco’s medium (IMDM; HiMedia, Mumbai, India) supplemented with 10% Mesen-fetal bovine serum (FBS) (Invitrogen, California, USA) and mouse-specific growth factors (IL-3, 5 ng/ml; IL-6, 12.5 ng/ml; and SCF, 12.5 ng/ml; Peprotech, NJ, USA), and treated with sodium nitroprusside (SNP; Alexis, NY, USA) at various concentrations and time intervals as indicated in the results section. Likewise, treatment of sorted LSK-CD34^−^ cells with various pharmacological reagents has been described in the respective results section. The viability of cells was >98% as determined by Trypan blue dye exclusion method.

### Isolation of murine lineage-negative cells and sorting of LSK-CD34^−^ and LSK-CD34^+^ cells

Murine lineage negative (Lin^−^) cells were isolated from the bones of Ptprc (CD45.1) mice. The mice were sacrificed and the hind limbs (femur and tibia) were dissected out. The bone marrow was flushed into IMDM supplemented with 5% FBS, and the MNCs were isolated by density gradient centrifugation (HiSep™ Hi Media, Mumbai, India). For the isolation of Lin^−^ cells the MNCs were first incubated with biotin-labeled anti-mouse lineage antibody cocktail (Additional file 1: Table S1) prepared from biotin mouse lineage panel (BD Pharmingen, New Jersey, USA) and then the antibody-bound lineage-positive cells were depleted using Dynabeads® biotin binder (Invitrogen, Carlsbad, California, USA).

For sorting of LSK-CD34^−^ and LSK-CD34^+^ cells, the MNCs were incubated with antibodies (Additional file 1: Table S1) against lineage markers, Sca-1, c-Kit, and CD34. Cells were washed to remove unbound antibodies and LSK-CD34^−^ and LSK-CD34^+^ cells were sorted on FACS ARIA II (Becton Dickinson, New Jersey, USA).

### Phenotypic analysis of hematopoietic progenitors

To analyze hematopoietic progenitors, murine lineage negative (Lin^–^) cells were isolated from mouse BM by immuno-magnetic separation as mentioned above. These cells were then cultured for 3 days with or without SNP. Cells were harvested and stained with antibodies against Lineage markers, c-Kit, Sca-1, IL-7R (CD127), and FcR (CD16/32) (Additional file 1: Table S1). Cells were washed and acquired on FACS Canto II (Becton Dickinson). Common myeloid populations (CMP; Lin^−^c-Kit^+^Sca-1^−^FcR^low^CD34^+^) and common lymphoid population (CLP; Lin^−^IL-7R^+^c-Kit ^low^Sca-1^low^) were analyzed as per the gating strategy shown in Additional file 2 (Figure S5).

### Human samples

CB and MBPL samples were collected from local hospitals after obtaining informed consent with the compliance of the institutional review board (Institutional ethical committee (IEC) and Institutional Committee for Stem Cell Research (IC-SCR) of the NCCS) according to the Declaration of Helsinki. Consenting procedures were also approved by the IC-SCR of the NCCS. MNCs were isolated from CB and MBPL by density gradient separation using HiSep (HiMedia). MNCs (5 × 10^6^) were suspended in IMDM supplemented with 10% FBS and human-specific growth factors (IL-3, 5 ng/m; IL-6, 12.5 ng/ml; and SCF, 12.5 ng/ml; Peprotech, NJ, USA). They were treated with SNP (100 μM) for 3 days and analyzed on a flow cytometer.

### Flow cytometric analysis

The immuno-stained cells were acquired on FACS Canto II ((Becton Dickinson). Doublets were excluded by FSC-H × FSC-W and SSC-H × SSC-W. Appropriate isotype control antibodies were used for each antibody. Single flurochrome-stained cells were used for compensation. All flow-qualified antibodies were purchased from BD Biosciences (San Jose, CA, USA) or eBioscience (San Diego, CA, USA), unless mentioned otherwise. Specific clones of various antibodies used are listed in Additional file 1 (Table S1). Data were analyzed using BD FACS DIVA as well as BD Flow Jo software.

### EdU labeling assay

To analyze the frequency of proliferating LSK-CD34^−^ and LSK-CD34^+^ cells, EdU (5-ethynyl-2’-deoxyuridine) labeling assay was performed using Click-iT® EdU Alexa Fluor® 647 Flow Cytometry Assay Kit (Invitrogen, Carlsbad, California, USA) as per the manufacturer’s instructions. Briefly, lineage-negative hematopoietic cells were treated with SNP (100 μM) for 3 days. Cells without the addition of SNP were used as controls. Cells were pulsed with EdU for 2 h and then harvested. The cells were fixed and permeabilized using the permeabilization buffer provided in the kit, and EdU reaction cocktail was added. The cells were stained using antibodies to Sca-1, c-Kit, and lineage markers, and acquired on a flow cytometer. The frequency of EdU^+^ cells in LSK-CD34^−^ and LSK-CD34^+^ HSCs was determined using BD FACS DIVA software.

### Nitrite estimation

To estimate nitrite production, the conditioned media from various cultures were collected, spun to remove debris, and the amounts of nitrites formed were estimated using Griess reagent. Conditioned media was mixed with an equal amount of 1% Griess reagent (1% sulphanilamide in 5% phosphoric acid, 0.1% naphthyl ethylene diamine dihydrochloride) and incubated for 10 min at room temperature. OD_540_ was measured using a spectrophotometer. A standard graph was prepared using various concentrations of NaNO_2_.

### Gene expression analysis

Cells were lysed in lysis buffer, and mRNA was isolated using the Dynabeads® mRNA DIRECT™ Purification Kit (Invitrogen) according to the manufacturer’s instructions. C-DNA was prepared using Superscript III^RT^ enzyme (Invitrogen). Samples without reverse transcriptase were used to detect genomic DNA contamination. Primers were designed using Primer-3 software V0.4.0 (http://primer3.ut.ee). Primer sequences are listed in Additional file 1 (Table S2). Quantitative RT-PCRs were performed using the Platinum® SYBR® Green (DSS Takara Bio India Pvt. Ltd., New Delhi, India) and analyzed on the ABI 7500 fast system (Applied Biosystems, Foster City, CA, USA). Gene expression was normalized with the β-actin. Relative gene expression was calculated as 2^ΔCt^.

### Gene silencing

siRNA against *c-Myb* was synthesized in vitro using a Silencer® siRNA Cocktail Kit (RNase III) (Invitrogen, California, USA) as per the manufacturer’s instruction. Briefly, using *c-Myb*-specific primers having a T7-promoter leader (8 nucleotides) sequence (Additional file 1: Table S3), a *c-myb*-specific stretch of cDNA was amplified from LSK-CD34^−^ cells. Amplicons were again amplified using T7 promoter primer. The sense and antisense siRNA templates were transcribed by T7 RNA polymerase and the resulting RNA transcripts were hybridized to create dsRNA. RNAse III was used to produce siRNAs (12–30 base pairs long) which were further purified. The resulting *c-myb* siRNA or *c-jun* siRNA (Santa Cruz Biotech, TX, USA) were transfected into sort-purified LSK-CD34^−^ cells using Dharmafect reagent (Thermo Scientific, MA, USA) in a 1:1 ratio. Mock transfected cells were used as controls. Efficiency of silencing of these SiRNA was determined by qRT-PCR using *c-Myb-* and *c-Jun*-specific primers. Cells were incubated for 12–18 h and then treated with SNP for 12 h. Levels of *c-Jun, c-Myb*, and *Cd34* mRNA were analyzed by qRT-PCR.

### In vivo transplantation assays

The CD45.1 and CD45.2 congenic chimera mouse model was used. For primary transplantation, lineage-depleted HSCs (CD45.1) from various cultures were harvested and 1 × 10^6^ cells admixed with 1 × 10^5^ freshly isolated CD45.2 cells were intravenously infused into lethally irradiated (9.5 Gy, two split doses given 4 h apart using γ-radiation from a Co^60^ source) recipients (CD45.2). The level of chimerism in the peripheral blood of the recipients was assessed after 4 and 16 weeks of transplantation. Engraftment by donor HSCs (LSK-CD34^+^ and LSK-CD34^−^) in the bone marrow of recipients was analyzed at 16 weeks post-transplant.

For secondary transplantation, the engrafted donor cells were sorted from the MNCs isolated from the tibia and femur bones of the primary recipients, and 5 × 10^5^ sorted donor cells were infused into irradiated secondary recipients (CD45.2). The donor cell chimerism in the peripheral blood of secondary recipients was analyzed 4 and 16 weeks post-transplantation. Engraftment of donor HSCs (LSK-CD34^+^ and LSK-CD34^−^) in the bone marrow of recipients was analyzed at 16 weeks post-transplant.

### Statistical analyses

Results were analyzed by one-way repeated-measures analysis of variance using the software Sigma Stat (Jandel Scientific Corporation, San Rafael, CA, USA) for all the experiments. P ≤ 0.05 was considered significant. Results are expressed as mean value ± SEM.

## Results

### NO donors increase the frequency of LSK-CD34^+^ HSCs

**Fig. 1:**
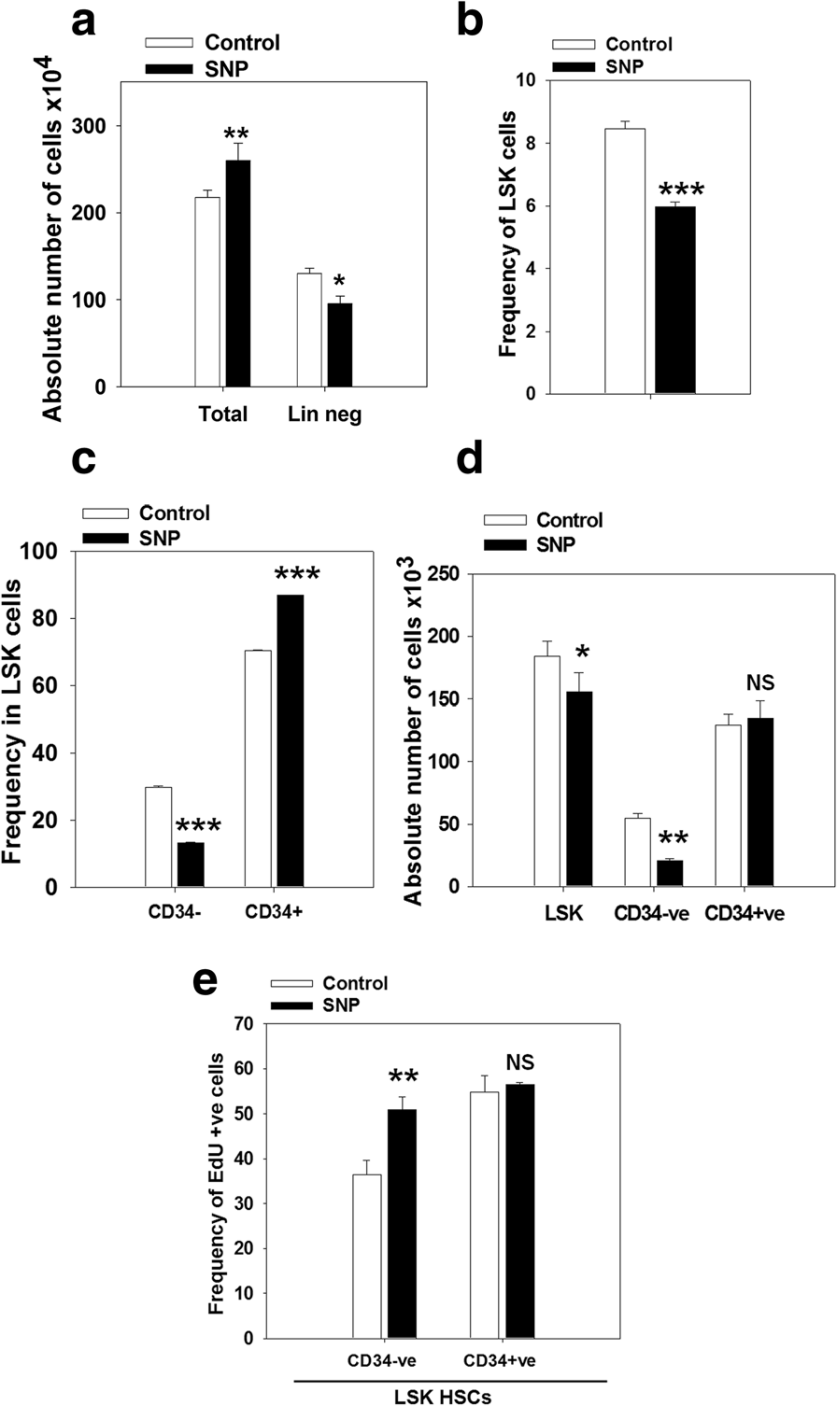
Sodium nitroprusside (*SNP*) increases the frequency of LSK-CD34^+^ hematopoietic stem cells (*HSCs*). Murine lineage-negative (*Lin* ^*−*^) bone marrow cells were exposed to 100 μM of SNP for 3 days and the output cells were enumerated and analyzed by flow cytometry. **a** Absolute numbers of hematopoietic cells and lineage-negative cells generated in the cultures are depicted. **b** Frequency of LSK HSCs in the output cells. **c** Frequency of CD34^−^ and CD34^+^ HSCs in the gated LSK population is illustrated. **d** Graph shows absolute numbers of LSK, LSK-CD34^−^, and LSK-CD34^+^ HSCs. **e** The frequency of EdU^+^ cells in the gated LSK-CD34^−^ and LSK-CD34^+^ population is illustrated. The data are represented as mean ± SEM. *N* = 3. *p ≤ 0.05, **p ≤ 0.01, ***p ≤ 0.001. Also see Additional file 3 (Figure S1). *NS* not significant

To analyze the effect of NO on murine HSCs, lineage-negative (Lin^−^) cells isolated from murine bone marrow (6–8 weeks old) were treated with 100 μM of SNP for 3 days. At concentrations up to 200 μM, SNP did not show any cytotoxicity (data not shown). The total number of hematopoietic cells significantly increased after treatment with SNP, but the number of Lin^−^ cells decreased (Fig. 1a; Additional file 3: Figure S1a). Flow cytometry analysis of the output cells (Additional files 1 and 3: Table S1 and Figure S1b) showed that SNP treatment significantly reduced the frequencies and total numbers of LSK-HSCs (Fig. 1b and d; Additional file 3: Figure S1a and c). A concomitant increase in the frequency of LSK-CD34^+^ HSCs and a decrease in the frequency of LSK-CD34^−^ HSCs were seen (Fig. 1c). The ratio of CD34^+^:34^−^ LSK-HSC was reversed as compared to the control cells and the input populations (Additional file 3: Figure S1d). The absolute number of LSK-CD34^−^ cells decreased significantly, but the absolute numbers of LSK-CD34^+^ cells did not change appreciably (Fig. 1d), suggesting that the increase in LSK-CD34^+^ cells was perhaps occurring at the expense of LSK-CD34^−^ cells.

To address this issue, SNP-treated or untreated Lin^−^ cells were subjected to EdU-labeling assay [18]. We found that the percentage of EdU^+^ cells among the LSK-CD34^−^ HSCs was significantly higher in SNP-treated cells, as compared to the control cells, but the percentage of EdU^+^ LSK-CD34^+^ cells was similar in both sets (Fig. 1e; Additional file 3: Figure S1e). These data show that the LSK-CD34^−^ HSCs proliferate in response to NO. This led us to hypothesize that the increase in the LSK-CD34^+^ population in response to NO is likely to be related to the acquisition of CD34 expression by LSK-CD34^−^ cells, rather than expansion of LSK-CD34^+^ cells.

### NO donors upregulate CD34 expression in LSK-CD34^−^ HSCs

**Fig. 2:**
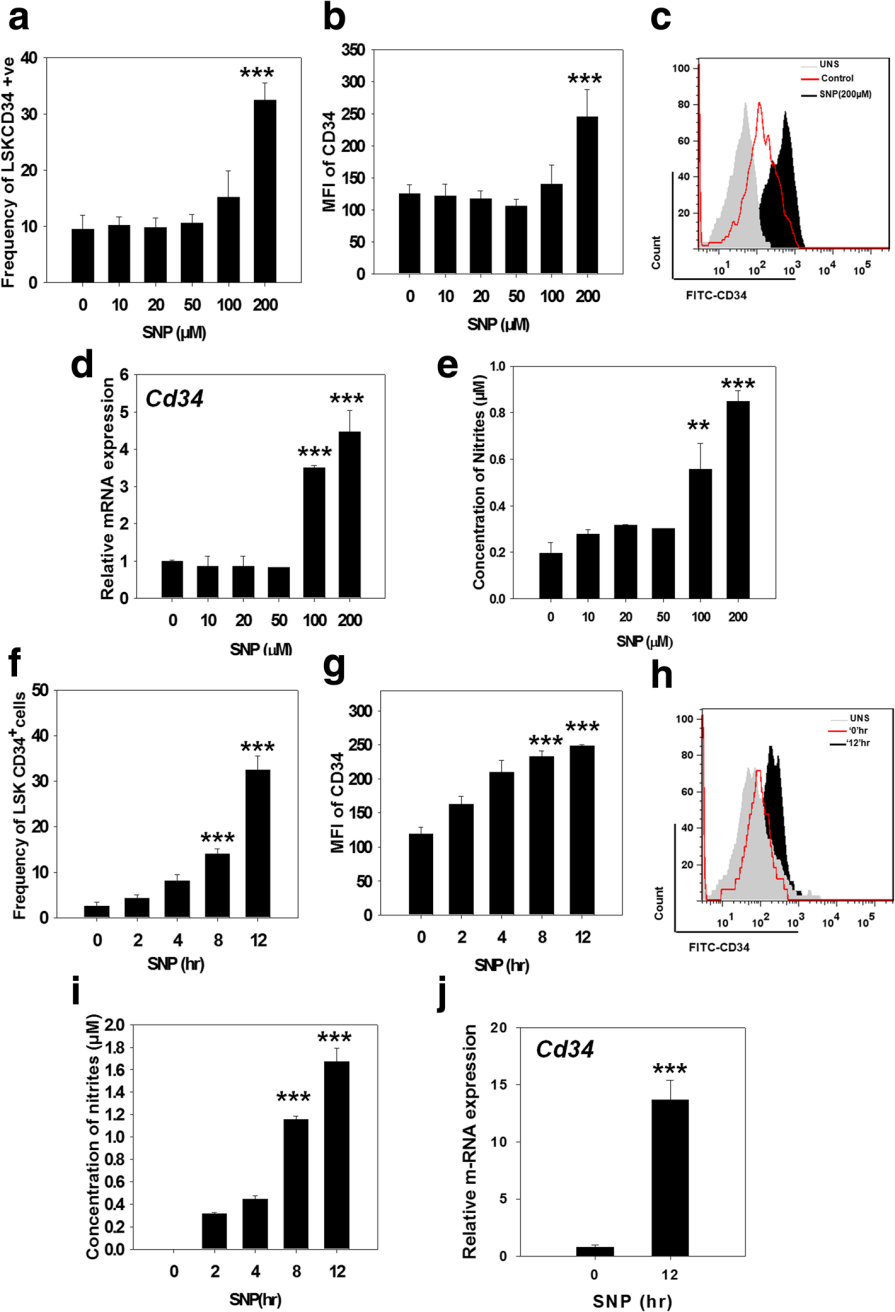
Sodium nitroprusside (*SNP*) upregulates surface expression and mRNA of CD34 in the LSK-CD34^−^ HSCs. (**a-e**) Sort-purified LSK-CD34^−^ HSCs were exposed to various concentrations of SNP for 12 h. The cells were analyzed for the surface expression of CD34 by flow cytometry and for *Cd34* mRNA expression by qRT-PCR. **a** Frequency of LSK-CD34^+^ cells formed in response to various concentrations of SNP. **b**, **c** The graph and the flow panel show mean fluorescence intensity (*MFI*) of CD34 fluorescence. **d** Dose-dependent effect of SNP on *Cd34*-specific mRNA levels in the treated cells. **e** The amounts of nitrites formed in the conditioned medium of cells. (**f-j**) Sort-purified LSK-CD34^−^ cells were exposed to 200 μM SNP for various time periods. The cells were analyzed for the surface expression of CD34 by flow cytometry. **f** The time-dependent increase in the frequency of LSK-CD34^+^ cells in response to SNP treatment is shown. **g**, **h** The graph and the flow panel show MFI of CD34 fluorescence. **i** Amounts of nitrites formed in the conditioned medium of the cells. **j** Expression of *Cd34*-specific mRNA in the cells treated with 200 μM SNP for 12 h. The data are represented as mean ± SEM. *N* = 3. **p ≤ 0.01, ***p ≤ 0.001. Also see Additional file 4 (Figure S2)

To test the hypothesis that the NO-mediated increase in LSK-CD34^+^ cells is due to the acquisition of CD34 expression by CD34^−^ HSCs, sort-purified LSK-CD34^−^ HSCs were exposed to various concentrations of SNP for 12 h and analyzed on a flow cytometer. We found that SNP had no effect on the frequency of LSK-CD34^+^ cells at concentrations up to 50 μM (Fig. 2a). The frequency started rising at a concentration of 100 μM, but the most significant increase in the frequency of LSK-CD34^+^ cells and the highest mean fluorescence intensity (MFI) of CD34 were seen at 200 μM SNP (Fig. 2a, b and c). The treated cells were also subjected to qRT-PCR analysis to examine whether the upregulation of CD34 was also happening at the transcriptional level (Additional file 1: Table S2). We found that levels of *Cd34* mRNA also remained steady up to 50 μM SNP, but increased significantly with 100 and 200 μM SNP (Fig. 2d). This increase in the frequency of CD34^+^ cells and increase in *Cd34* mRNA closely matched with the increase in the concentration of nitrites formed in the conditioned medium, showing that the release of NO was necessary for the observed effects (Fig. 2e).

SNP was then used to treat LSK-CD34^−^ HSCs at a concentration of 200 μM for various time periods to determine the time-dependent effect of NO on CD34 expression. The frequency of LSK-CD34^+^ cells and their MFI increased steadily with an increase in the duration of SNP treatment, and at 12 h this increase became highly significant (Fig. 2f, g and h). This increase closely correlated with the increase in the nitrite concentration in their conditioned medium (Fig. 2i). Based on these data, the LSK-CD34^−^ cells were treated with 200 μM SNP for 12 h and were subjected to qRT-PCR analyses. We found a significant upregulation of C*d34*-spcific mRNA expression in the treated cells (Fig. 2j).

We also examined the effect of SNP on sort-purified LSK-CD34^+^ (ST-HSCs) cells and found that SNP also upregulated the surface expression and MFI of CD34 in these cells (Additional file 4: Figure S2a, b and c). An increase in the *Cd34*-specific mRNA was also seen in these cells with a treatment of 200 μM SNP for 12 h (Additional file 4: Figure S2d).

Collectively, these data confirmed that LSK-CD34^−^ HSCs are the primary cellular targets of NO action with respect to CD34 expression. All further experiments were performed on sorted LSK-CD34^−^ cells treated with 200 μM SNP for 12 h, unless stated otherwise.

### Pure NO is required for the induction of CD34 mRNA and surface expression

**Fig. 3:**
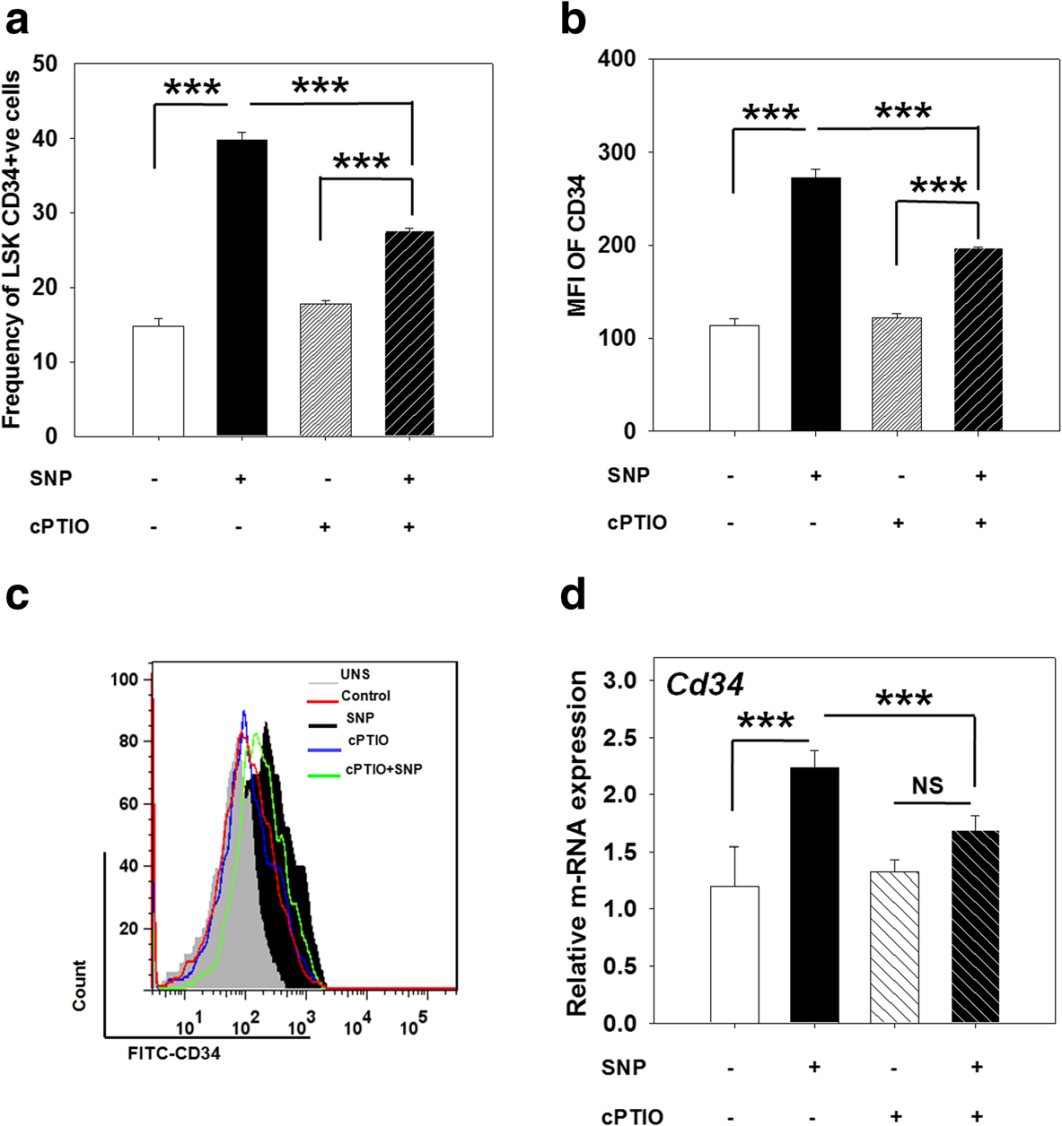
Addition of the nitric oxide scavenger c-PTIO abolishes sodium nitroprusside (*SNP*)-mediated upregulation of CD34 expression. Sort-purified LSK-CD34^−^ cells were exposed to 200 μM SNP for 12 h with or without the addition of 100 μM c-PTIO. The cells were analyzed for CD34 surface expression by flow cytometry, and *Cd34*-specific mRNA was quantified by qRT-PCR. **a** Graph shows frequency of LSK-CD34^+^ cells formed in cultures. **b**, **c** The graph and the flow panel show the mean fluorescence intensity (*MFI*) of CD34 fluorescence. **d** Relative expression of *Cd34*-specific mRNA in the treated cells. The data are represented as mean ± SEM. *N* = 3. ***p ≤ 0.001. Also see Additional file 5 (Figure S3). *NS* not significant

It is known that pharmacological agents such as SNP may also generate by-products other than NO. To determine whether NO was involved in the upregulation of surface expression of CD34, the sorted LSK-CD34^−^ cells were incubated with SNP in the presence of 100 μM c-PTIO (2-(4-carboxyphenyl)-4,4,5,5-tetramethylimidazoline-1-oxyl-3-oxide), which is a specific scavenger of NO. We found that in the presence of c-PTIO, the SNP-mediated increase in the surface expression and MFI of CD34 was significantly reduced (Fig. 3a, b and c). Similarly, the SNP-mediated increase in *Cd34* mRNA levels was also prevented by the addition of c-PTIO (Fig. 3d). These data point towards a direct role of NO in the upregulation of surface expression of CD34 in the HSCs.

Since pharmacological agents may have off-target effects, the sorted LSK-CD34^−^ cells were exposed to two other NO donors, namely 3-morpholinosydnonimine (SIN-1) and diethylenetriamine/nitric oxide adduct (DETA NONOate; NOC-18) for 12 h. We found that both compounds increased the surface expression and MFI of CD34 in a dose-dependent manner (Additional file 5: Figure S3a, b, c and d), clearly establishing that CD34 is indeed a direct target of NO action.

### Nitric oxide-mediated upregulation of CD34 is dependent on de novo mRNA and protein synthesis

**Fig. 4:**
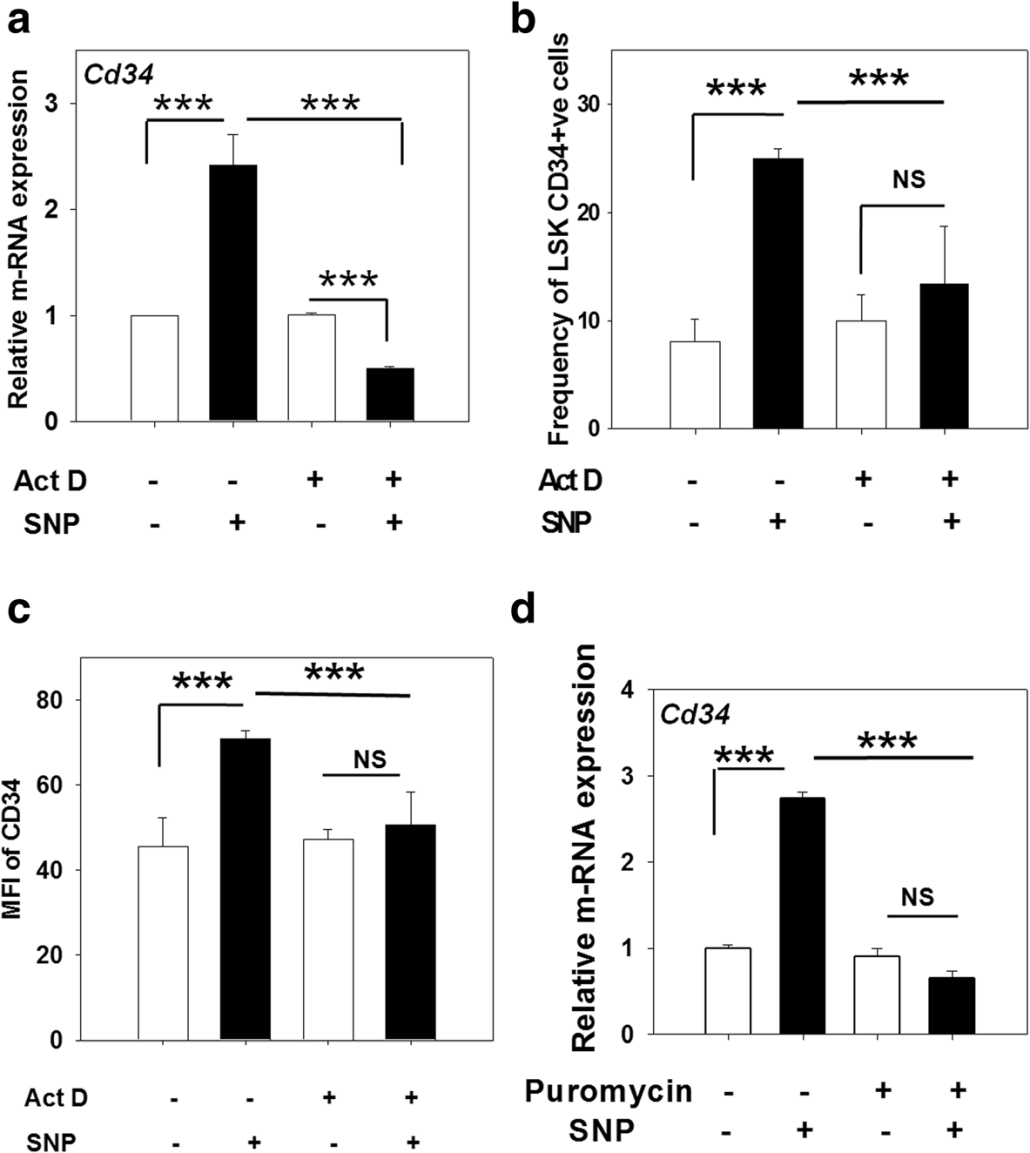
Upregulation of CD34 expression by sodium nitroprusside (*SNP*) is dependent on de novo mRNA and protein synthesis. Sort-purified LSK-CD34^−^ cells were treated with 200 μM SNP for 12 h with or without the addition of 5 μg/ml actinomycin D (*Act D*). The cells were subjected to qRT-PCR and flow cytometry analyses. **a** Relative expression of Cd34-specific mRNA in the treated cells. **b** The frequency of LSK-CD34^+^ cells formed in the cultures. **c** Effect of ActD on the mean fluorescence intensity (*MFI*) of CD34 fluorescence is depicted. **d** Sort-purified LSK-CD34^−^ cells were treated with 200 μM SNP for 12 h with or without the addition of pruromycin (20 μg/ml). The cells were subjected to qRT-PCR analyses. Relative expression of *Cd34*-specific mRNA in the treated cells is illustrated. The data are represented as mean ± SEM. *N* = 3. ***p ≤ 0.001. *NS* not significant

To elucidate whether the induction of CD34 expression in response to NO required de novo synthesis of mRNA, the sort-purified LSK-CD34^−^ HSCs were treated with actinomycin D (ActD; 5 μg/ml) and SNP or with ActD alone for 12 h and were subjected to qRT-PCR analysis. As shown in Fig. 4a, the SNP-mediated increase in *Cd34*-specific mRNA in the LSK CD34^−^ cells was inhibited in the presence of ActD, indicating the requirement of a de novo mRNA synthesis for the process. A similar effect was also seen at the protein level (Fig. 4b and c). These data reveal that NO-mediated upregulation of CD34 requires de novo mRNA synthesis.

We then examined whether NO-mediated upregulation of *Cd34* needs de novo protein synthesis. The sort-purified LSK-CD34^−^ HSCs were incubated for 12 h with SNP in the presence or absence of puromycin (20 μg/ml), an inhibitor of translation initiation. We found that puromycin inhibited the NO-mediated upregulation of *Cd34* mRNA (Fig. 4d), showing that NO-mediated upregulation of *Cd34* requires de novo protein synthesis as well.

### NO-induced CD34 expression is independent of cGMP, but involves S-nitrosylation of proteins

**Fig. 5:**
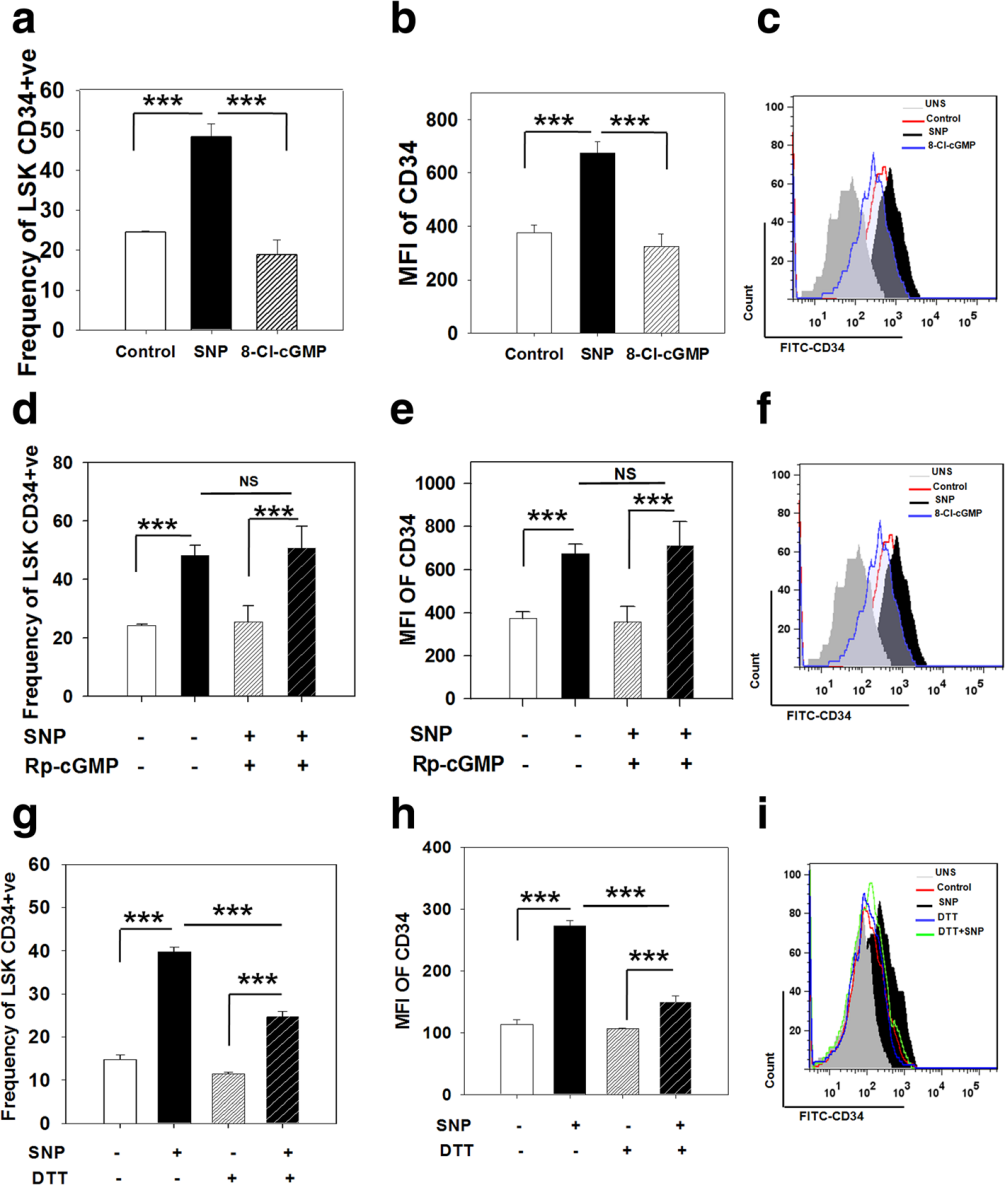
NO-mediated CD34 expression is independent of the activation of the cGMP signaling pathway, but depends on S-nitrosylation of proteins. Sort-purified LSK-CD34^−^ cells were exposed to 200 μM sodium nitroprusside (*SNP*) or 100 μM 8-Cl-cGMP, a membrane-permeable cGMP analogue, for 12 h. The cells were analyzed by flow cytometry. **a** The frequency of LSK-CD34^+^ cells generated in the culture is graphically illustrated. **b**, **c** The graph and the flow panel show mean fluorescence intensity (*MFI*) of CD34 fluorescence. Sort-purified CD34^−^ cells were exposed to 200 μM SNP in the presence or absence of 100 μM Rp-cGMP, a specific inhibitor of cGMP-dependent protein kinase, for 12 h. The cells were subjected to flow cytometry analysis. **d** The frequency of LSK-CD34^+^ cells generated in the cultures is illustrated. **e**, **f** The graph and the flow panel show the MFI of CD34 fluorescence. Sort-purified CD34^−^ cells were exposed to 200 μM SNP in the presence or absence of 200 μM DTT for 12 h. **g** The frequency of LSK-CD34^+^ cells generated in the cultures is depicted. **h**, **i** The graph and the flow panel show the MFI of CD34 fluorescence. The data are represented as mean ± SEM. *N* = 3. ***p ≤ 0.001. Also see Additional file 5 (Figure S3). *NS* not significant

NO is known to mediate its effects through the activation of sGC, which leads to an increase in cGMP [19]. To determine whether the SNP-induced increase in CD34 expression was dependent on cGMP, the effect of the membrane-permeable cGMP analogue, 8-Cl-cGMP, on membrane expression of CD34 was examined. After exposure of sorted LSK-CD34^−^ cells to 8-Cl-cGMP (100 μM) for 12 h, CD34 surface expression and its MFI remained unaltered (Fig. 5a, b and c). Next, we examined the effect of Rp-cGMP, a specific inhibitor of cGMP-dependent protein kinase. Sorted LSK-CD34^−^ cells were pre-incubated with 100 μM Rp-cGMP for 30 min before the addition of SNP. Addition of Rp-cGMP did not alter the SNP-mediated upregulation of CD34 or its MFI (Fig. 5d, e and f). Expression of *Vegf-a-*specific mRNA in BMSCs directly treated with 8-Cl-cGMP and BMSCs treated with SNP in the presence of Rp-cGMP was used as a positive control to demonstrate the activity of Rp-cGMP and 8-Cl-cGMP, respectively (Additional file 5: Figure S3e–f). Collectively, these data show that NO-mediated upregulation of CD34 is independent of intracellular cGMP.

NO can alter a wide variety of cellular functions by S-nitrosylation of proteins [20]. To determine whether the induction of CD34 expression by NO involves such a mechanism, sorted LSK-CD34^−^ cells were incubated for 30 min with 200 μM DTT before the addition of SNP. We found that the SNP-mediated increase in surface expression and MFI of CD34 was significantly reduced by the addition of DTT (Fig. 5 g, h and i), suggesting that NO exerts its effect on CD34 by S-nitrosylation of proteins.

### NO upregulates CD34 gene expression via *c-Myb* and *c-Jun*

**Fig. 6:**
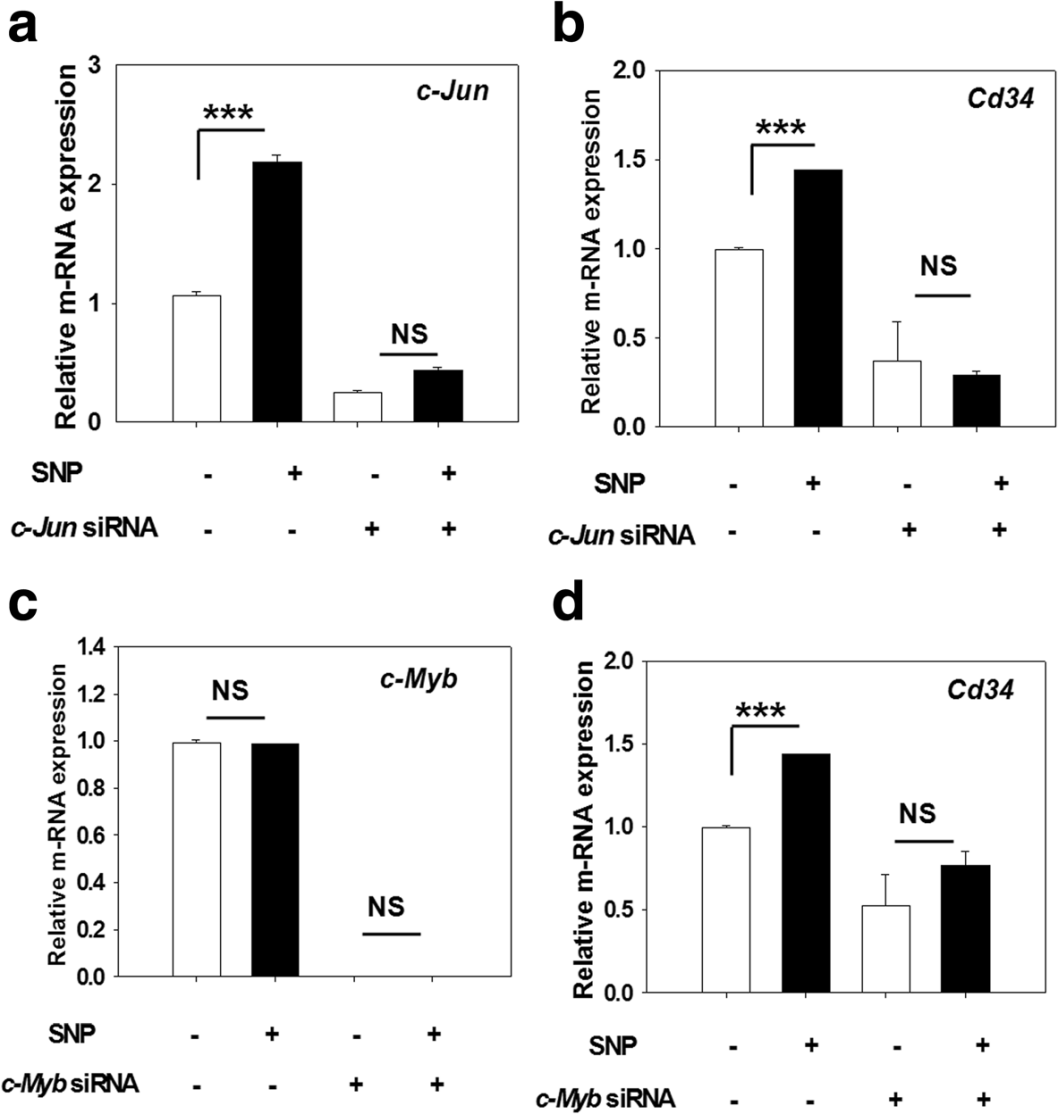
NO-mediated upregulation of CD34 involves *c-Myb* and *c-Jun.* Sort-purified LSK-CD34^−^ cells were transfected with siRNA to *c-Jun* and *c-Myb*. Mock transfected cells were used as controls. After 18 h post-transfection, the cells were exposed to 200 μM sodium nitroprusside (*SNP*) for 12 h and then subjected to qRT-PCR analyses. Relative expression of *c-Jun*-specific mRNA (**a**) and *Cd34*-specific mRNA (**b**) in the cells transfected with *c-Jun*-specific siRNA, with or without the treatment with SNP, is depicted. Relative expression of *c-Myb*-specific mRNA (**c**) and *Cd34*-specific mRNA (**d**) in the cells transfected with *c-Myb*-specific siRNA, with or without the treatment with SNP, is depicted. ***p ≤ 0.001. *NS* not significant

Transcription factors (TFs) such as c-Myb and c-Jun are known to play an important role in stem cell biology and are also known to be regulated by NO in various cell systems [21–23]. First, we determined whether SNP has any effect on their expression in the HSCs. The LSK-CD34^−^ cells showed a basal level expression of both these TFs, and SNP treatment upregulated the expression of *c-Jun* in LSK-CD34^−^ HSCs, but did not affect c*-Myb* expression (Fig. 6a and c). Aliquots of LSK-CD34^−^ HSCs were transfected separately with *c-Jun-* and *c-Myb*-specific siRNA (Additional file 1: Table S3) 18 h before the treatment with SNP, and were subjected to qRT-PCR analyses. We found that the NO-mediated upregulation of *Cd34* mRNA was significantly affected in the cells transfected with siRNA to c-Jun and c-Myb (Fig. 6b and d), showing that the process involved both TFs. The effect of silencing of *c-Jun* was more striking, as compared to that seen with *c-Myb* silencing, indicating that *c-Jun* plays a more crucial role in the process.

### NO-induced CD34 expression has age-dependent implications in the functionality of HSCs

**Fig. 7:**
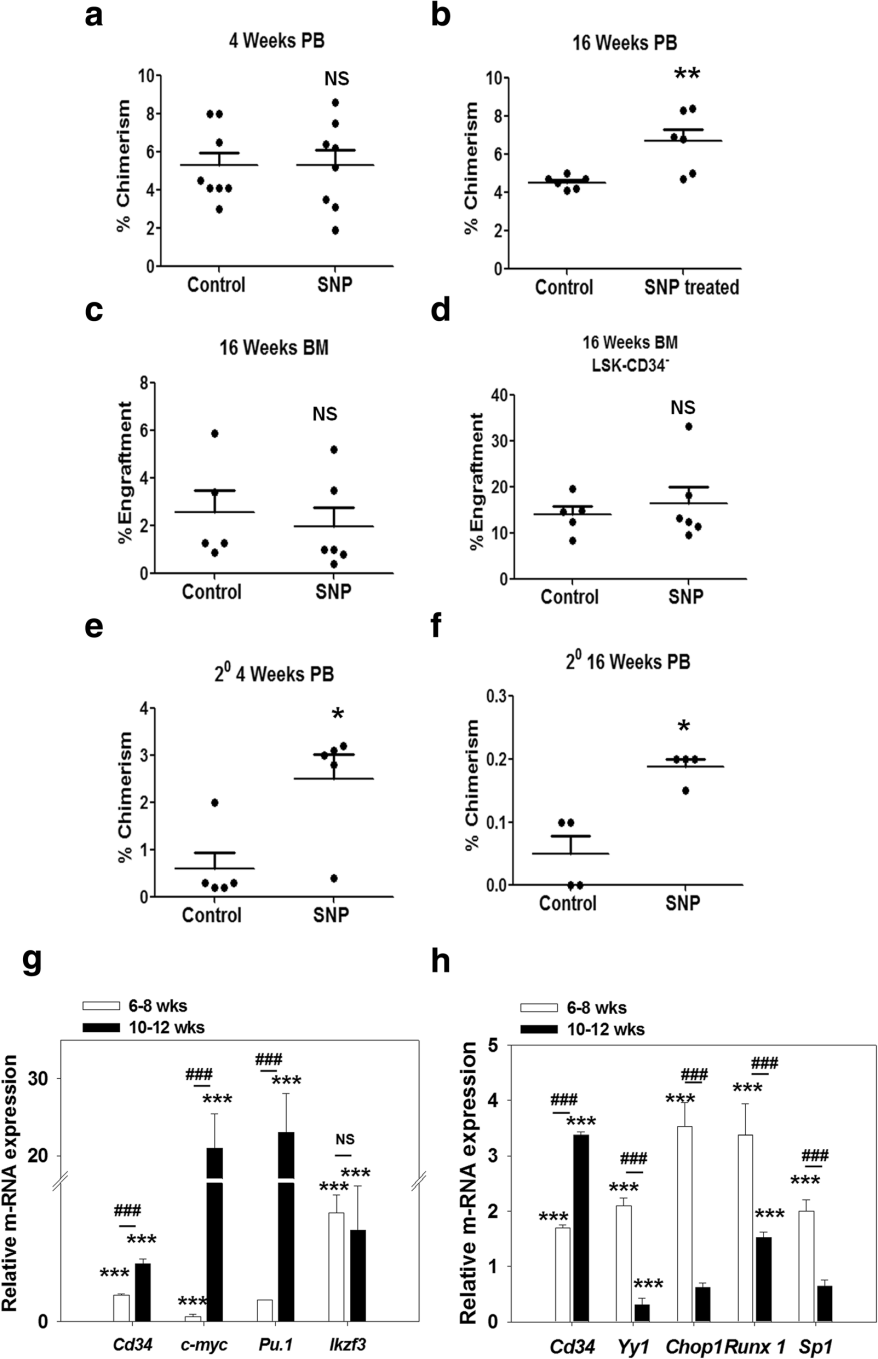
NO-induced CD34 expression has contrasting age-dependent effects on the HSC functionality**.** Lin^−^ cells isolated from juvenile mice (6–8 weeks; CD45.1) were treated or not with 200 μM sodium nitroprusside (*SNP*) for 3 days and infused into irradiated recipients (8–10 weeks; CD45.2); 7–8 mice were used per group. The level of engraftment was analyzed by flow cytometry analysis of peripheral blood (*PB*) and bone marrow (*BM*). Percentage chimerism by the donor cells in PB of recipients at 4 weeks (**a**) and 16 weeks (**b**) post-transplant is depicted. Percentage engraftment of total donor cells (**c**) and LSK CD34^−^ LT-HSCs (**d**) in the BM of recipients is illustrated. The engrafted donor cells were sorted from the bone marrow of primary recipients and infused into irradiated secondary recipients. The percentage chimerism by these primary engrafted donor cells was analyzed by flow cytometry in the PB of secondary recipients. Percentage chimerism produced by the primary engrafted cells in the PB of secondary recipients at 4 weeks (**e**) and 16 weeks (**f**) post-transplant is shown; 4–5 secondary recipients were used per group. Sort-purified LSK-CD34^−^ cells from juvenile (6–8 weeks) and adult (10–12 weeks) mice were treated with 200 μM SNP for 3 days (**g**) or 12 h (**h**), and the cells were subjected to qRT-PCR analyses. Relative expression of *Cd34* and various transcription factors is illustrated (means ± SEM; *N* = 3). *p ≤ 0.05, **p ≤ 0.01, ***p ≤ 0.001, between control and SNP-treated sets; ^###^ *p* ≤ 0.001, SNP-treated juvenile versus SNP-treated adult HSCs. Also see Additional file 6 (Figure S4). *NS* not significant

CD34^+^ HSCs from juvenile mice (6 weeks old) have been shown to possess engraftment ability [24, 25]. We therefore determined the consequences of upregulated CD34 expression in the HSCs on their in vivo functionality (Additional file 6: Figure S4a). The Lin^−^ cells (6–8 weeks old) were treated with SNP for 3 days and infused into irradiated recipients (8–10 weeks old). At 4 weeks post-transplant, the level of chimerism in the peripheral blood (PB) of the recipients was comparable in the control and SNP-treated sets (Fig. 7a; Additional file 6: Figure S4b). However, at 16 weeks a significant increase in the level of chimerism was observed with the SNP-treated cells as compared to controls (Fig. 7b). No lineage bias was seen in the regenerated blood cells at 4 weeks post-transplant in either set (Additional file 6: Figure S4c). At 16 weeks post-transplant, the percentage of myeloid cells was significantly lower in the SNP-treated set as compared to the control set, but the percentage of B and T cells was similar in both (Additional file 6: Figure S4d). Surprisingly, analysis of BM performed at 16 weeks post-transplant showed that the percentage of total donor cells and LSK-CD34^−^ HSCs were not significantly different between the two groups (Fig. 7c and d). The engrafted donor cells from the bone marrow of primary recipients were sorted and infused into irradiated secondary recipients to assess their long-term functionality. We found that, in secondary hosts, the cells sorted from primary recipients that had received SNP-treated cells showed significantly higher engraftment at both 4 and 16 weeks post-transplant (Fig. 7e and f). These data suggest that the NO-mediated increase in CD34 expression is perhaps a reflection of self-renewal in juvenile HSCs.

Previous reports have shown that HSCs collected from adult mice (12 weeks) treated with NO donors possess reduced engraftment ability, suggesting that NO has a detrimental effect on HSC functionality [26]. In sharp contrast, we found that SNP treatment of juvenile HSCs boosted their engraftment ability. To determine the effect of SNP on the engraftment ability of adult HSCs, adult Lin^−^ cells (10–12 weeks) treated with SNP for 3 days were infused into irradiated hosts and their engraftment levels were monitored at 4 and 16 weeks post-transplant. We found that the level of chimerism at 4 weeks post-transplant was comparable in both control and SNP-treated sets, but at 16 weeks post-transplant the SNP-treated adult cells showed significantly lower chimerism in the PB of the recipients (Additional file 3: Figure S5a and b). No lineage bias was observed in the regenerated blood cells formed in the PB of the recipients at both 4 and 16 weeks post-transplant showing that although the overall engraftment ability of SNP-treated cells was compromised, the engrafted HSCs could give rise to multi-lineage hematopoiesis under the influence of a local micro-environment (Additional file 3: Figure S5c and d). BM analysis performed at 16 weeks also showed that the number of LSK-CD34^−^ LT-HSCs was significantly lower in the SNP-treated set, although the total number of engrafted donor cells was comparable in both control and SNP-treated sets (Additional file 3: Figure S5e and f). These data clearly demonstrated that NO has an age-dependent effect on HSC functionality.

### NO upregulates CXCR4 surface expression in both juvenile and adult HSCs

The lack of difference in the engraftment levels shown by control and SNP-treated cells in the BM of recipients as compared to the significant differences in percentage chimerism in the PB in recipients prompted us to examine the reason behind this discrepancy. CXCR4 plays an important role in the retention of HSCs in the BM milieu. Furthermore, NO has been shown to induce CXCR4 expression in human CD34^+^ HSCs [27]. We therefore examined whether NO also increases CXCR4 expression in murine HSCs. We found that both juvenile and adult LSK-CD34^−^ and LSK-CD34^+^ HSCs showed an enhanced mRNA and surface expression of CXCR4 in response to NO (Additional file 7: Figure S6a and b). These data show that perhaps this NO-mediated enhanced CXCR4 expression enabled the SNP-treated adult as well as juvenile HSCs to home and accumulate in the BM of recipients at a similar level. These data demonstrate that reduced chimerism exhibited by SNP-treated adult HSCs in the PB of recipients was not related to loss of CXCR4 expression, but was perhaps related to their inability to form blood cells as effectively as SNP-treated juvenile HSCs, suggesting loss of self-renewal in them.

### Molecular mechanism behind age-dependent contrasting effect of NO on HSC functionality involves differential expression of transcription factors

The differential effect of NO on the HSC functionality prompted us to examine whether this was related to differential expression of self-renewal with regard to commitment-related TFs in the HSCs in an age-dependent manner. To examine this, the sorted LSK-CD34^−^ cells collected from 6–8 weeks old (juvenile HSCs) and 10–12 weeks old (adult HSCs) mice were treated with SNP for 3 days and were analyzed by qRT-PCR. The adult HSCs showed very high expression of *c-Myc* and *PU.1* as compared to the juvenile HSCs (Fig. 7 g). The levels of *Ikzf3* (Ikaros) was similar in both the sets. Overexpression of c-Myc leads to a loss of self-renewal ability of the HSCs at the expense of differentiation [28] and *PU.1* is a known myeloid commitment marker [29]. Consistent with these results, the frequency of common myeloid progenitors (CMP) in the output population of SNP-treated adult Lin^−^ cells was significantly higher as compared to that in SNP-treated juvenile Lin^−^ cells. The frequency of common lymphoid progenitor (CLP) was similar in both the sets (Additional file 7: Figure S6c and d). Interestingly, the level of *Cd34* was significantly higher in the adult HSCs as compared to the juvenile HSCs (Fig. 7 g). These data show that NO induces a commitment program in the adult HSCs but not in the juvenile HSCs.

We then decided to examine whether such differential regulation was also seen with respect to other TFs. We short-listed the TFs that bind to the CD34 promoter (TF-bind software) and are regulated by NO in other experimental systems. We then selected the ones that are relevant in the context of hematopoiesis. The sorted LSK-CD34^−^ cells treated or not with SNP for 12 h were subjected to qRT-PCR analyses and the expression of selected genes was quantified. The relative levels of *Cd34* were also assessed. As observed in the earlier experiment, the SNP-mediated increase in *Cd34* expression in adult HSCs was much higher as compared to that in juvenile HSCs (Fig. 7 h). Also, the genes related to self-renewal such as *Yy1*, *Chop1*, and *Runx1* [30–32] were significantly upregulated in juvenile HSCs as compared to the adult HSCs. *Sp1* is known to positively regulate the CD34 promoter [33]. Surprisingly, its expression was higher in juvenile HSCs as compared to the adult HSCs. Since *Cd34* expression was significantly lower in these cells, it appears that the effect of *Sp1*was countered by some unknown mechanisms. Other genes such as *Nfya, Atf1*, *Vbp1* and *c-Myb* [34–38] were not affected by NO in either cells (Additional file 7: Figure S6e). NO upregulated *c-Jun* expression at comparable levels in both juvenile and adult HSCs. The level of *Trp53* (p53) [39] in SNP-treated adult HSCs was significantly lower as compared to that seen in SNP-treated juvenile HSCs.

Collectively, these data show that the age-dependent contrasting effect of NO on HSC functionality is due to induction of a self-renewal program in juvenile HSCs and a commitment process in adult HSCs.

### NO also exerts age-dependent effects on human HSCs

To examine whether NO also exerts age-dependent effects on the human HSCs, we performed preliminary experiments with CB- and MBPL-derived cells to obtain a proof of principle. CB- and MBPL-derived MNCs were treated with SNP (100 μM) for 3 days and the output cells were subjected to qRT-PCR and flow cytometry analyses. SNP treatment resulted in a significant increase in *CD34*-specific mRNA in both juvenile and adult cells (Additional file 8: Figure S7b). The increase in CD34 mRNA in adult cells was strikingly high. Flow analysis revealed that the frequency of CD34^+^38^−^ HSCs increased significantly in SNP-treated CB cells (Additional file 8: Figure S7a and c), but decreased significantly in SNP-treated MPBL cells. A similar result was also obtained when the fold-change in the HSC numbers over their input was determined (Additional file 8: Figure S7d). Since human CB- or BM-derived CD34^+^38^−^ HSCs are known to have engraftment capacity [40–42], our data show that treatment of CB cells, but not of adult cells, leads to a significant increase in HSCs having engraftment potential in them. These preliminary data suggest that NO has an age-dependent effect on CD34 surface expression, and perhaps also functionality, of human HSCs as well. This aspect needs to be studied in greater detail.

## Discussion

**Fig. 8:**
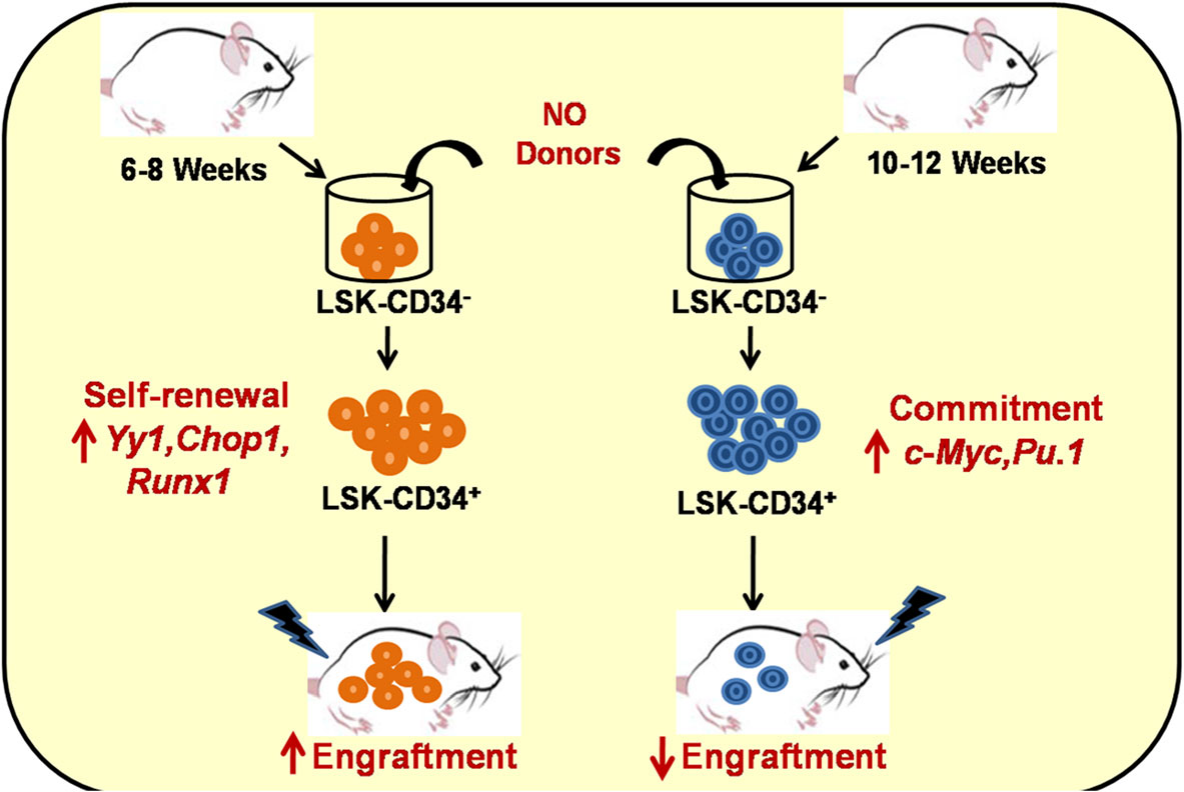
Graphical summary. *NO* nitric oxide

Success of transplantation critically depends on the quality and functionality of donor HSCs. Therefore, use of pharmacological agents to improve engraftment of donor HSCs is being aggressively pursued. In the present study, we examined the effect of NO donors on the engraftment ability of murine HSCs. We also elucidated the molecular mechanisms involved in the process. Here, we show for the first time that NO induces CD34 expression in LSK-CD34^−^ HSCs and this has a contrasting age-dependent effect on their functionality (Fig. 8).

In a murine system, CD34 antigen expression is considered as a marker of short-term engrafting HSCs (ST-HSCs). However, previous reports have demonstrated that the expression of CD34 on murine HSCs is context-dependent, and has two implications: it could either be an indication of progressive commitment and differentiation giving rise to ST-HSCs or it could indicate an activation status of LT-HSCs, as shown in 5-fluorouracil-treated mice [43]. It has also been shown that CD34^+^ HSCs collected from young mice (6–8 weeks old; juvenile HSCs) possess engraftment capacity, but the CD34^−^ do not, while in adult mice (>10 weeks old; adult HSCs) the situation is reversed [24]. These data show that CD34 expression has age- as well as context-dependent implications in murine hematopoiesis. In our study, we found that SNP-treated juvenile HSCs engraft more efficiently, whereas their adult counterparts do not. Thus, our data provide direct evidence for the context-dependent role of CD34 in functionality of murine HSCs.

In vitro exposure of murine BM cells (12 weeks old) to NO donors has been reported to induce myeloid commitment of HSCs. Similarly, treatment of adult mice with NO donors resulted in expansion of HSCs in their bone marrow, but these HSCs lost their long-term engraftment capacity [26]. Although these results were suggestive of an increase in ST-HSCs, whether LSK-CD34^−^ LT-HSCs acquired CD34 expression in response to NO and contributed to the ST-HSC population, or whether LSK-CD34^+^ cells themselves proliferated in response to NO, was not investigated. Also, previous studies did not distinguish between the effects of NO on juvenile as compared to adult HSCs. Such a distinction is important since CD34 expression has been shown to have development-specific implications in the functionality of murine HSCs [24].

In the present study, we identified murine CD34 and LSK-CD34^−^ cells as the molecular and cellular targets, respectively, of NO signaling. We demonstrated that NO induces CD34 surface expression and *Cd34* mRNA expression in LSK-CD34^−^ cells in a time- and dose-dependent manner. This was also supported by a strong correlation between the upregulation of CD34 and the nitrite concentrations in the medium (an indirect measure of NO). These data clearly establish that the NO-mediated increase in LSK-CD34^+^ cells is related to acquisition of CD34 expression by the LSK-CD34^−^ HSCs, rather than proliferation of LSK-CD34^+^ HSCs.

Very few reports describe the mechanisms involved in the regulation of CD34 expression in murine HSCs. The transcription start sites of both murine and human CD34 genes contain consensus binding sites for several transcription factors, three of which, myb, ets and mzf-1, have been shown to affect CD34 expression [44–46]. *Sp1* has been shown to regulate murine CD34 at the transcriptional level [33]. To the best of our knowledge, ours is the first report showing that NO regulates CD34 expression in murine HSCs at both the transcriptional and the translational levels.

NO is known to regulate several TFs [47], including *c-Myb* and *c-Jun*. Both these TFs play an important role in HSC proliferation and are also known to regulate CD34 expression in various cell systems [37, 48, 49]. Using siRNA-mediated gene silencing, we demonstrated that the NO-mediated upregulation of CD34 is dependent on *c-Myb* as well as *c-Jun.* Silencing of c-Jun had a stronger effect on NO-mediated CD34 expression in the LSK CD34^−^ HSCs, as compared to silencing of *c-Myb*, suggesting that *c-Jun* plays a crucial role in the process. The milder effects seen with silencing of *c-Myb* are consistent with the work published by Krause et al. [50]. Upregulation of mitochondrial biogenesis has been shown to parallel upregulation of CD34 in murine HSCs [51]. Whether NO upregulates mitochondrial biogenesis in the murine HSCs remains to be seen.

The most important finding in this study was that the NO-mediated upregulation of CD34 in juvenile HSCs (6–8 weeks) boosted their engraftment capacity. In sharp contrast, such an upregulation of CD34 in the adult HSCs (10–12 weeks) significantly reduced their engraftment capacity. These differences in the engraftment capacities of juvenile and adult CD34^+^ HSCs are similar to previous reports showing that CD34^+^ HSCs from juvenile mice, but not from adult mice, possess engraftment capacity [24, 25]. High levels of c-Myc are known to compromise the self-renewal ability of HSCs [28].We found that the NO-mediated upregulation of CD34 was accompanied by a strong upregulation of *c-Myc* in adult HSCs, but not in juvenile HSCs. Similarly, the expression of *Pu.1*, a myeloid commitment marker, was highly upregulated only in the adult HSCs. These data indicate that NO-induced CD34 expression is accompanied by an induction of the differentiation program in adult HSCs.

The contrasting functionality observed in juvenile compared to adult HSCs could also be correlated with the differential regulation of some important TFs by NO in the two types of HSCs. The expression of specific TFs selected for their binding to the CD34 promoter, their involvement in NO signaling in various systems, and their importance in the hematopoietic system was examined in NO-treated HSCs from juvenile and adult mice. *Yy1*, a multifunctional polycomb group (PcG) transcription factor, has been shown to mark phenotypically defined and functional LT-HSCs and is also known to interfere with the commitment process [30]. Consistent with these reports, *Yy1* was found to be significantly upregulated by NO in juvenile HSCs, but not in adult HSCs. The regulation of *Cd34* transcription requires *Sp1* [33]. We found that NO upregulated *Sp1* in both types of HSCs, but in juvenile HSCs the levels of *Sp1* were significantly higher. However, the level of *Cd34* in NO-treated juvenile HSCs was significantly lower as compared to that in NO-treated adult HSCs. TF-bind software-based analysis showed that *Yy1* binds to the *Cd34* promoter, but its role in the regulation of *Cd34* has not been elucidated. YY1 is a ubiquitously distributed transcription factor that is involved in repressing and activating a diverse number of promoters. It would be interesting to determine whether it negatively regulates CD34 expression, thereby countering the positive regulatory effects of *Sp1* on *Cd34* in juvenile cells. Yet another TF that was strongly upregulated by NO in juvenile HSCs was *Chop1*. It is a basic leucine-zipper protein induced by a variety of stimuli, including NO [52], that evokes cellular stress responses. On the other hand, deletion of its homologous TF, C/EBP, has been shown to affect self-renewal of HSCs [31]. It is thus possible that, in the juvenile HSCs, *Chop1* acts as a self-renewal factor and boosts the engraftment ability of the HSCs. Consistent with previous reports showing *Runx-1* as a marker for long-term engrafting HSCs [32], we found that *Runx1* levels in NO-treated juvenile HSCs were significantly higher as compared to their adult counterparts. Thus, high levels of *Yy1*, *Chop1*, and *Runx1*, coupled with a comparatively lower level of *Cd34*, may have resulted in the self-renewal and increased functionality of juvenile HSCs. Our findings emphasize the age-specific influences of NO in filling an important gap in the existing knowledge about its role in hematopoiesis.

The surface expression of CXCR4 on HSCs plays an important role in their homing, engraftment, and retention in the bone marrow of recipients [53]. NO has been shown to enhance CXCR4 expression in human CD34^+^ HSCs collected from adult (BM and mobilized PB) as well as neonatal sources (CB). Taking into consideration the differences in engraftment shown by NO-treated juvenile and adult HSCs, a possibility exists that this may have been due to age-dependent effects of NO on CXCR4 expression in murine HSCs. However, we found that the treatment of juvenile as well as adult murine HSCs with NO resulted in a strong upregulation of CXCR4 on their surface, ruling out the possibility that the differential effect of NO on their transplantation ability could be related to differential effects on CXCR4 surface expression. Treatment of murine HSCs with SNP has been shown to abrogate PGE2-mediated upregulation of CXCR4 [10]. However, we found that SNP increases CXCR4 in both adult and juvenile HSCs. It is possible that a combined presence of SNP and PGE2 negatively regulates CXCR4. Our data showing compromised engraftment of SNP-treated adult HSCs in spite of elevated CXCR4 expression suggest that a mere upregulation of CXCR4 surface expression cannot be taken as a reliable indicator of engraftment ability of the HSCs.

Our data could have relevance in clinical transplantation. Use of pharmacological compounds to enhance homing and engraftment of HSCs has been widely attempted for clinical transplantation. Such manipulation becomes important when the HSCs are either fewer in numbers (fetal liver, CB) or possess compromised quality (aged donors). Our murine data, taken together with the preliminary data generated with CB and MBPL cells, show that NO exerts age-dependent effects on HSC functionality.

In a wider perspective, our data point towards a possibility that several other pharmacological reagents may also have such contrasting effects on the functionality of HSCs from developmentally distinct sources, and therefore HSC age may be considered as an important parameter while screening various compounds for their potential use in HSC manipulation.

## Conclusions

This study demonstrates novel age-dependent contrasting effects of NO on HSC functionality and suggests that HSC age may be an important parameter for screening of various compounds for their use in manipulation of HSCs.

## Additional files

Additional file 1: Table S1. Antibodies. **Table S2.** List of gene-specific primer sequence used for qRT-PCR analyses. **Table S3.** Sequence of siRNA primers used for in vitro siRNA synthesis. (DOCX 20 kb)
Additional file 2: Figure S5. Treatment of adult HSCs with NO donor reduces their engraftment ability. The donor cell chimerism in the PB of recipients at 4 weeks (a) and 16 weeks (b) is depicted. (c, d) Lineage commitment in regenerated hematopoietic cells in the PB of recipients at 4 and 16 weeks post-transplant is illustrated. After 16 weeks post-transplant the mice were sacrificed and their BM cells were analyzed for percentage engraftment by the donor cells. Percentage engraftment of total donor cells (e) and percentage LSK-CD34^−^ LT-HSCs (f) in the recipients’ BM at 16 weeks post-transplant is illustrated. **p* ≤ 0.05, ****p* ≤ 0.001. Also see Fig. 7. (TIF 761 kb)
Additional file 3: Figure S1. Fold-change in the number (a) and the frequency (c) of various cells formed in the cultures treated or not with 200 μM SNP for 3 days is illustrated. Data represent means ± SEM of three independent experiments performed. (b) Flow panel illustrates the gating strategy used for flow cytometry analyses of HSC subpopulations. (d) Graph depicts the ratio of LSK-CD34^+^ HSCs to LSK-CD34^−^ HSCs in the starting population and in the cells generated after 3 days of incubation with or without the addition of 200 μM SNP. (e) Flow panel illustrates the gating strategy used for EdU labeling experiment. ****p* ≤ 0.001. Also see Fig. 1. (TIF 4360 kb)
Additional file 4: Figure S2. SNP also increases surface expression and mRNA levels of CD34 in LSK-CD34^+^ cells. Sort-purified LSK-CD34^+^ cells were treated with various concentrations of SNP for 12 h and the cells were analyzed on a flow cytometer. The frequency of LSK-CD34^+^ HSCs formed in the cultures (a) and the MFI of CD34 fluorescence (b, c) is shown. (d) Sort-purified LSK-CD34^+^ cells were treated with 200 μM SNP for 12 h, and were subjected to qRT-PCR analyses. Relative expression of *Cd34*-specific mRNA expression is graphically illustrated. **p* ≤ 0.05, ***p* ≤ 0.01. Also see Fig. 2. (TIF 1397 kb)
Additional file 5: Figure S3. Effects of NO donors on the membrane expression of CD34. Sort-purified LSK-CD34^−^ cells were treated with various concentrations of SIN-1 and NOC-18 for 12 h. The increase in the frequency of LSK-CD34^+^ cells formed and MFI of CD34 fluorescence in the cells treated with SIN (a, b) and NOC-18 (c, d) are illustrated. ****p* ≤ 0.001. Also see Fig. 3. (e, f) Expression of *Vegf-a*-specific mRNA in BMSCs directly treated with 100 μM 8-Cl-cGMP and BMSCs treated with 100 μM SNP in the presence of 100 μM Rp-cGMP is depicted. The data show that 8-Cl-cGMP significantly increased the *Vegf-a* expression in the BMSCs (e). Similarly, SNP-mediated increase in *Vegf-a* expression was significantly inhibited by Rp-cGMP (f). These data show that both chemicals exhibit desired activity. **p* ≤ 0.05, ***p* ≤ 0.01, ****p* ≤ 0.001. Also see Fig. 5. (TIF 512 kb)
Additional file 6: Figure S4. Treatment of adult HSCs with NO donor does not induce lineage bias in regenerated blood cells. Lin^−^ cells from adult mice (10–12 weeks; CD45.1)) were treated with 200 μM SNP for 3 days and were infused into irradiated recipients (8–10 weeks; CD45.2). Six mice were used per group. Donor cell chimerism was determined by flow cytometry of recipients’ PB. (a) Experimental scheme is illustrated. (b) Gating strategy used to analyze donor cell chimerism in the PB of recipient mice. (c, d) Lineage commitment in regenerated hematopoietic cells in the PB of recipients at 4 and 16 weeks post-transplant is illustrated. Also see Fig. 7. (TIF 2833 kb)
Additional file 7: Figure S6. NO upregulates Cxcr4 expression in murine HSCs. (a) *Cxcr4*-specific mRNA expression and (b) MFI of CXCR4 surface expression in SNP-treated juvenile and adult HSCs is graphically illustrated. NO increases myeloid commitment of adult HSCs. (c) Gating strategy applied for flow analysis of common myeloid progenitors (CMP) and common lymphoid progenitors (CLP) is illustrated. (d) Frequencies of CMP and CLP formed in SNP-treated adult and juvenile Lin^−^ cells are graphically represented. Comparative transcription factor analyses of juvenile and adult HSCs treated with SNP. Sort-purified LSK-CD34^−^ cells from juvenile mice (6–8 weeks) and adult mice (10–12 weeks) were treated with 200 μM SNP for 12 h and subjected to qRT-PCR analysis. (e) Comparative gene expression of various transcription factors in the treated cells is shown (*N* = 3). **p* ≤ 0.05, ***p* ≤ 0.01, ****p* ≤ 0.001, comparison between control and SNP-treated sets; ^###^ *p* ≤0.001, comparison between SNP-treated juvenile versus SNP-treated adult HSCs. *NS* not significant. Also see Fig. 7. (TIF 2406 kb)
Additional file 8: Figure S7. NO exerts age-specific contrasting effects on human HSCs. (a) Gating strategy applied for human CD45^+^34^+^38^−^ HSCs is illustrated. (b) SNP-mediated increase in *CD34*-specific mRNA and (c) frequency of CD34^+^38^−^ HSCs in gated CD45^+^ population in SNP-treated CB and MBPL cells are graphically represented. (d) Fold-change in absolute numbers of CD45^+^34^+^38^−^ HSCs generated in cultures over input CD45^+^34^+^38^−^ HSC population is depicted. *N* = 3. **p* ≤ 0.05, ***p* ≤ 0.01, ****p* ≤ 0.001. (TIF 1949 kb)

## Abbreviations

ActD: Actinomycin D
BM: Bone marrow
CB: Cord blood
CLP: Common lymphoid populations
CMP: Common myeloid populations
ES: Embryonic stem
FBS: Fetal bovine serum
HSC: Hematopoietic stem cell
IL: Interluekin
IMDM: Iscove’s modified Dulbecco’s medium
iPS: Induced pluripotent stem
LSK: Lin^−^Sca-1^−^c-Kit^+^
LT-HSC: Long-term repopulating hematopoietic stem cell
MFI: Mean fluorescence intensity
MNC: Mononuclear cell
MPBL: Mobilized peripheral blood leukocytes
NO: Nitric oxide
NOS: Nitric oxide synthase
PB: Peripheral blood
SNP: Sodium nitroprusside
ST-HSC: Short-term repopulating hematopoietic stem cell
TF: Transcription factor
