# Supplementary material for: Nitric oxide has contrasting age-dependent effects on the functionality of murine hematopoietic stem cells
Source: Stem Cell Res Ther. 2016 Nov 22;7:171. doi: 10.1186/s13287-016-0433-x (PMC5120451; doi:10.1186/s13287-016-0433-x)
Supplement: Additional file 1: Table S1. — Antibodies. Table S2. List of gene-specific primer sequence used for qRT-PCR analyses. Table S3. Sequence of siRNA primers used for in vitro siRNA synthesis. (DOCX 20 kb) [file 13287_2016_433_MOESM1_ESM.docx]

| **Antigen** | **Flurochrome** | **Clone/Cat.no.** | **Distributor** |
| --- | --- | --- | --- |
| Lineage cocktail | V450 | 561301 | BD Biosciences |
| c-Kit | PECy7 | 2B8 | eBiosciences |
| CD34 | FITC | RAM11 | eBiosciences |
| CD184(CXCR4) | PE | 2B11/CXCR4 | BD Biosciences |
| Sca-1 | PE | D7 | BD Biosciences |
| IL-7R(CD127) | APCy7 | A7R34 | eBiosciences |
| Fc-R(CD16/32) | APC | 93 | eBiosciences |
| CD45.1 | APC | A20 | BD Biosciences |
| CD45.2 | FITC | 104 | BD Biosciences |
| CD3e | PEcy7 | 145-2C11 | eBiosciences |
| CD45R(B220) | eFluor 450 | RA3-6B2 | eBiosciences |
| CD11b(Mac-1-α) | PE | M1/70 | eBiosciences |
| Ly6G (Gr1) | PE | RB6-8C5 | eBiosciences |

**Supplementary data: Table S1: Antibodies**

**Table S2: List of gene-specific primer sequence used for qRT-PCR analyses**

| Genes | Gene ID | Primers | Sequences |
| --- | --- | --- | --- |
| *β- actin* | NM_007393.3 | Forward | 5´-GCCTTCCTTCTTGGGTATG-3´ |
|  |  | Reverse | 5´-CTAGGAGCCAGAGCAGTAAT-3´ |
| *Cd34* | BC006607.1 | Forward | 5'-CTTCTGCTCCGAGTGCCATATT -3' |
|  |  | Reverse | 5'- GCCAAGACCATCAGCAAACAC-3 |
| *Cxcr4* | NM_009911.3 | Forward | 5'-TCAGTGGCTGACCTCCTCTT-3' |
|  |  | Reverse | 5'-CTTGGCCTTTGACTGTTGGT-3 |
| *Ikzf3* | NM_011771.1 | Forward | 5'- AGCCGAGATGGGAAGTGAG-3' |
|  |  | Reverse | 5'- CTGATGGCGTTATTGATGG-3 |
| *c-Myc* | L00039.1 | Forward | 5'- TCTGGTAAGCTACCCCTTCCT -3 |
|  |  | Reverse | 5'- AGTTGTGCTGGTGAGTGGAG -3 |
| *Sp1* | NM_013672.2 | Forward | 5'- TCATGGATCTGGTGGTGATGGG -3 |
|  |  | Reverse | 5'- GCTCTTCCCTCACTGTCTTTGC -3 |
| *Runx 1* | NM_001111023.2 | Forward | 5'- ACTTCCTCTGCTCCGTGCTA -3 |
|  |  | Reverse | 5'- CGCGGTAGCATTTCTCAGTT -3 |
| *Trp53* | AY044188.1 | Forward | 5'- GCTGCTCCGATGGTGATG -3 |
|  |  | Reverse | 5'- AGTGTGATGATGGTAAGGATAGG -3 |
| *Hsf1* | NM_008296.2 | Forward | 5'- ATCCCTTTGGAAGGAGGTGT-3 |
|  |  | Reverse | 5'- TCTTTGGGGCTCCATTTGTG -3 |
| *Atf 1* | NM_007497.3 | Forward | 5'- TGCAGACCTACCAGATCCGT -3 |
|  |  | Reverse | 5'- TCTCCAGGCACTTCACGTAC -3 |
| *Vbp 1* | NM_011692.2 | Forward | 5'- GACGAGATTCTTACTGGCCG -3 |
|  |  | Reverse | 5'- GATCTCGAAGAAAGTCAAGG -3 |
| *Chop1* | NM_007837.4 | Forward | 5'- CCTAGCTTGGCTGACAGAGG -3 |
|  |  | Reverse | 5'- CTGCTCCTTCTCCTTCATGC -3 |
| *Nfya* | NM_001110832.1 | Forward | 5'- GCTCTGTGCCTGCTATCCAA -3 |
|  |  | Reverse | 5'- GCCGAGACTCATGGAGGTAT -3 |
| *c-Myb* | NM_001198914.1 | Forward | 5'- TCTTCTGCTCAAACCACTGG -3 |
|  |  | Reverse | 5'- CGGTAAAGGCTTTGAGGACA -3 |
| *c-Jun* | NM_010591.2 | Forward | 5'-GCCAACCTCAGCAACTTCAAC -3 |
|  |  | Reverse | 5'-ACGGTCTGCGGCTCTTCC -3 |
| *Yy1* | NM_009537.3 | Forward | 5'- ACCCTAAGCAACTGGCAGAA-3 |
|  |  | Reverse | 5'- GGTGTGCAGATGCTTTCTCA-3 |
| *Vegf-a* | NM_001025257.3 | Forward | 5'-CAGGCTGCTGTATAACGATGAA -3 |
|  |  | Reverse | 5'-CGCCTTGGCTTGTCACA -3 |

**Table S3: Sequence of siRNA /primers used for in vitro siRNA synthesis**

| **Sr. no.** | **siRNA** | **Cat no ./Primer Sequence** | **Distributor** |
| --- | --- | --- | --- |
| 1 | *c-Jun* | SC-29224 | Santacruz biotech |
| 2 | *c-Myb* | Forward Primer (For in vitro siRNA synthesis) 5’GGGCGGGTAAATATGGTCCGAAGCGTTG3’ | Eurofins |
|  |  | Reverse Primer (For in vitro siRNA synthesis)  5’ GGGCGGGTGGGAACGTGACTGGAGATGT 3’ | Eurofins |
