# Supplementary figures and images for: Nitric oxide has contrasting age-dependent effects on the functionality of murine hematopoietic stem cells
Source: Stem Cell Res Ther. 2016 Nov 22;7:171. doi: 10.1186/s13287-016-0433-x (PMC5120451; doi:10.1186/s13287-016-0433-x)

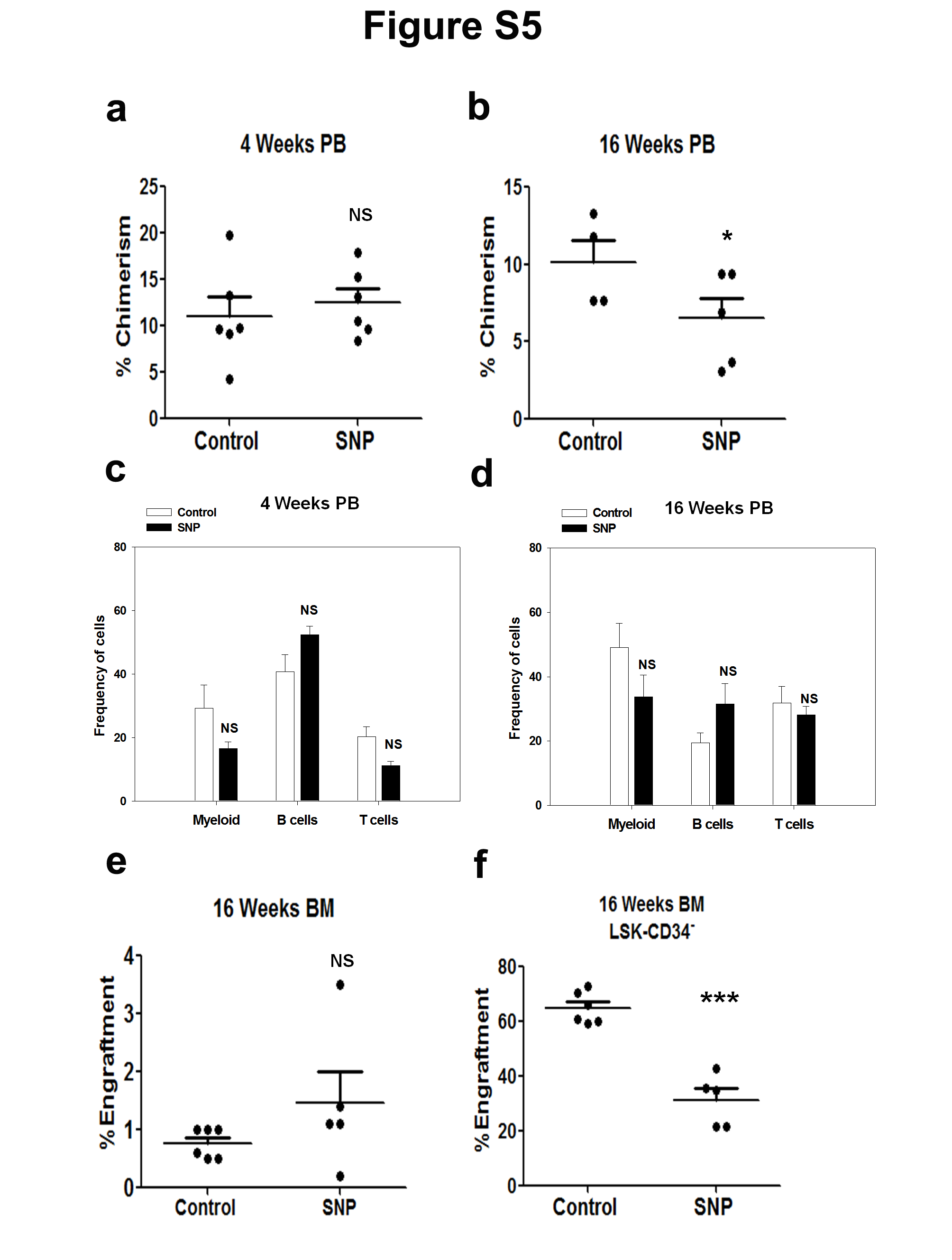

Supplement: Additional file 2: Figure S5. — Treatment of adult HSCs with NO donor reduces their engraftment ability. The donor cell chimerism in the PB of recipients at 4 weeks (a) and 16 weeks (b) is depicted. (c, d) Lineage commitment in regenerated hematopoietic cells in the PB of recipients at 4 and 16 weeks post-transplant is illustrated. After 16 weeks post-transplant the mice were sacrificed and their BM cells were analyzed for percentage engraftment by the donor cells. Percentage engraftment of total donor cells (e) and percentage LSK-CD34− LT-HSCs (f) in the recipients’ BM at 16 weeks post-transplant is illustrated. *p ≤ 0.05, ***p ≤ 0.001. Also see Fig. 7. (TIF 761 kb) [file 13287_2016_433_MOESM2_ESM.tif]

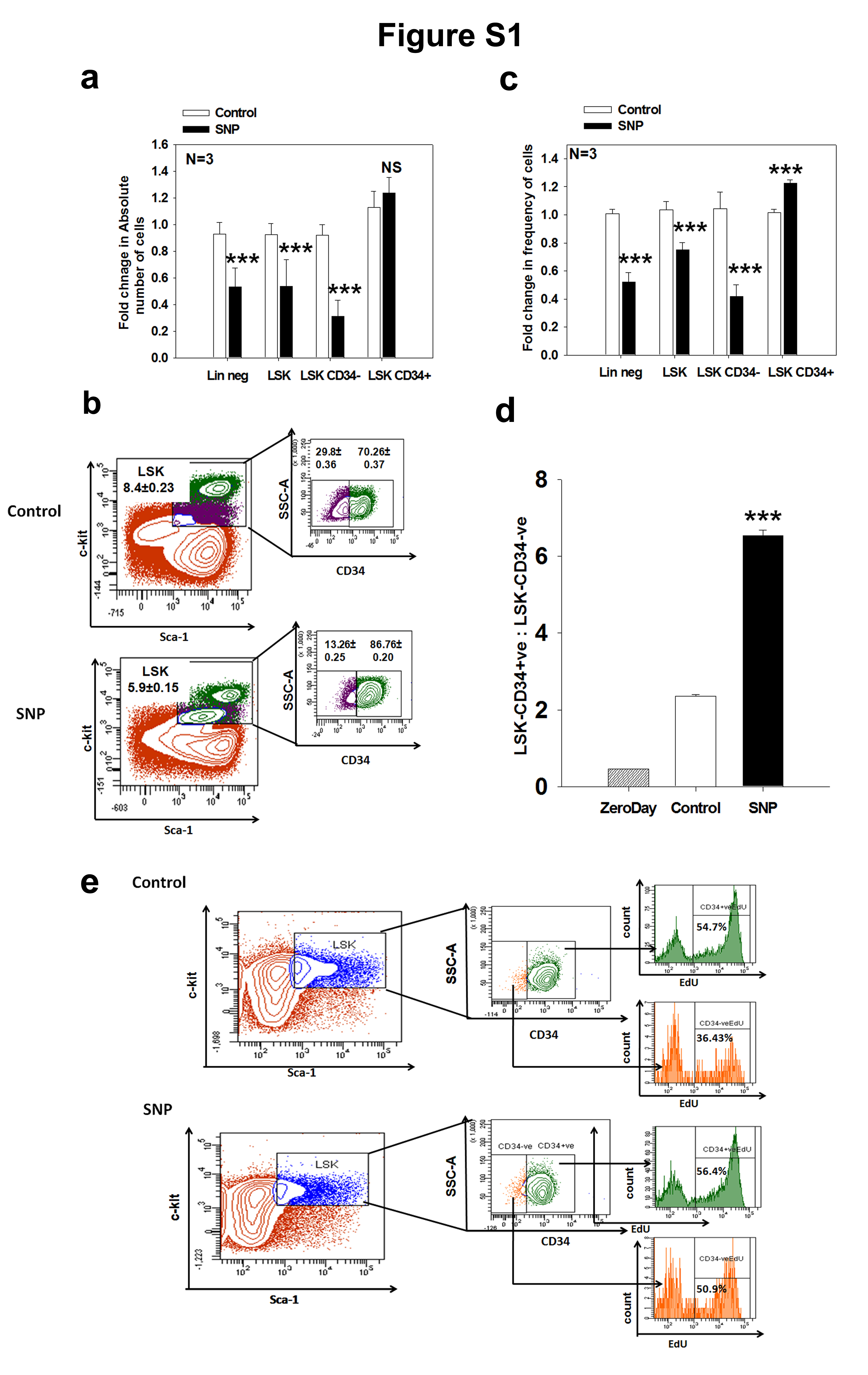

Supplement: Additional file 3: Figure S1. — Fold-change in the number (a) and the frequency (c) of various cells formed in the cultures treated or not with 200 μM SNP for 3 days is illustrated. Data represent means ± SEM of three independent experiments performed. (b) Flow panel illustrates the gating strategy used for flow cytometry analyses of HSC subpopulations. (d) Graph depicts the ratio of LSK-CD34+ HSCs to LSK-CD34− HSCs in the starting population and in the cells generated after 3 days of incubation with or without the addition of 200 μM SNP. (e) Flow panel illustrates the gating strategy used for EdU labeling experiment. ***p ≤ 0.001. Also see Fig. 1. (TIF 4360 kb) [file 13287_2016_433_MOESM3_ESM.tif]

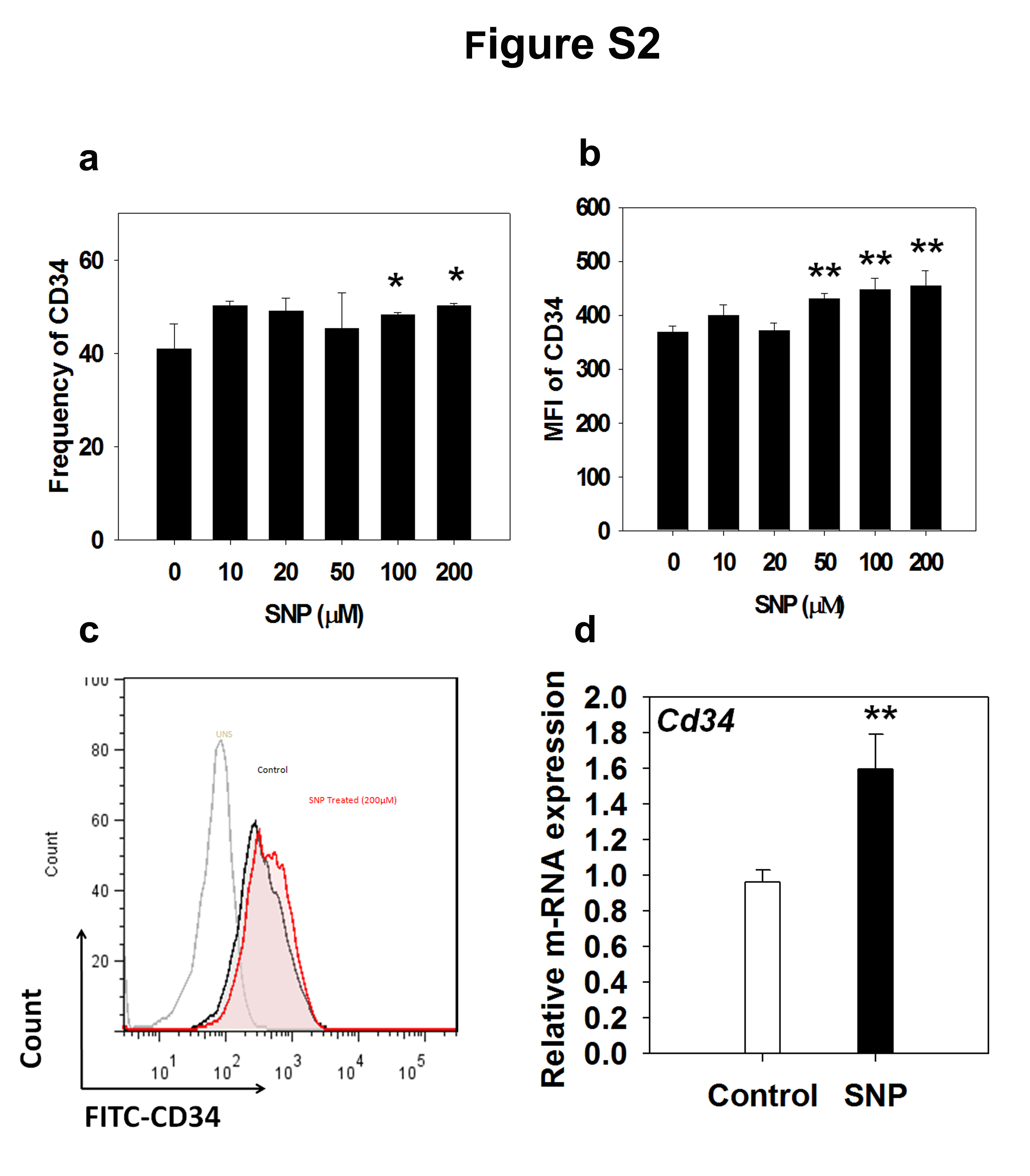

Supplement: Additional file 4: Figure S2. — SNP also increases surface expression and mRNA levels of CD34 in LSK-CD34+ cells. Sort-purified LSK-CD34+ cells were treated with various concentrations of SNP for 12 h and the cells were analyzed on a flow cytometer. The frequency of LSK-CD34+ HSCs formed in the cultures (a) and the MFI of CD34 fluorescence (b, c) is shown. (d) Sort-purified LSK-CD34+ cells were treated with 200 μM SNP for 12 h, and were subjected to qRT-PCR analyses. Relative expression of Cd34-specific mRNA expression is graphically illustrated. *p ≤ 0.05, **p ≤ 0.01. Also see Fig. 2. (TIF 1397 kb) [file 13287_2016_433_MOESM4_ESM.tif]

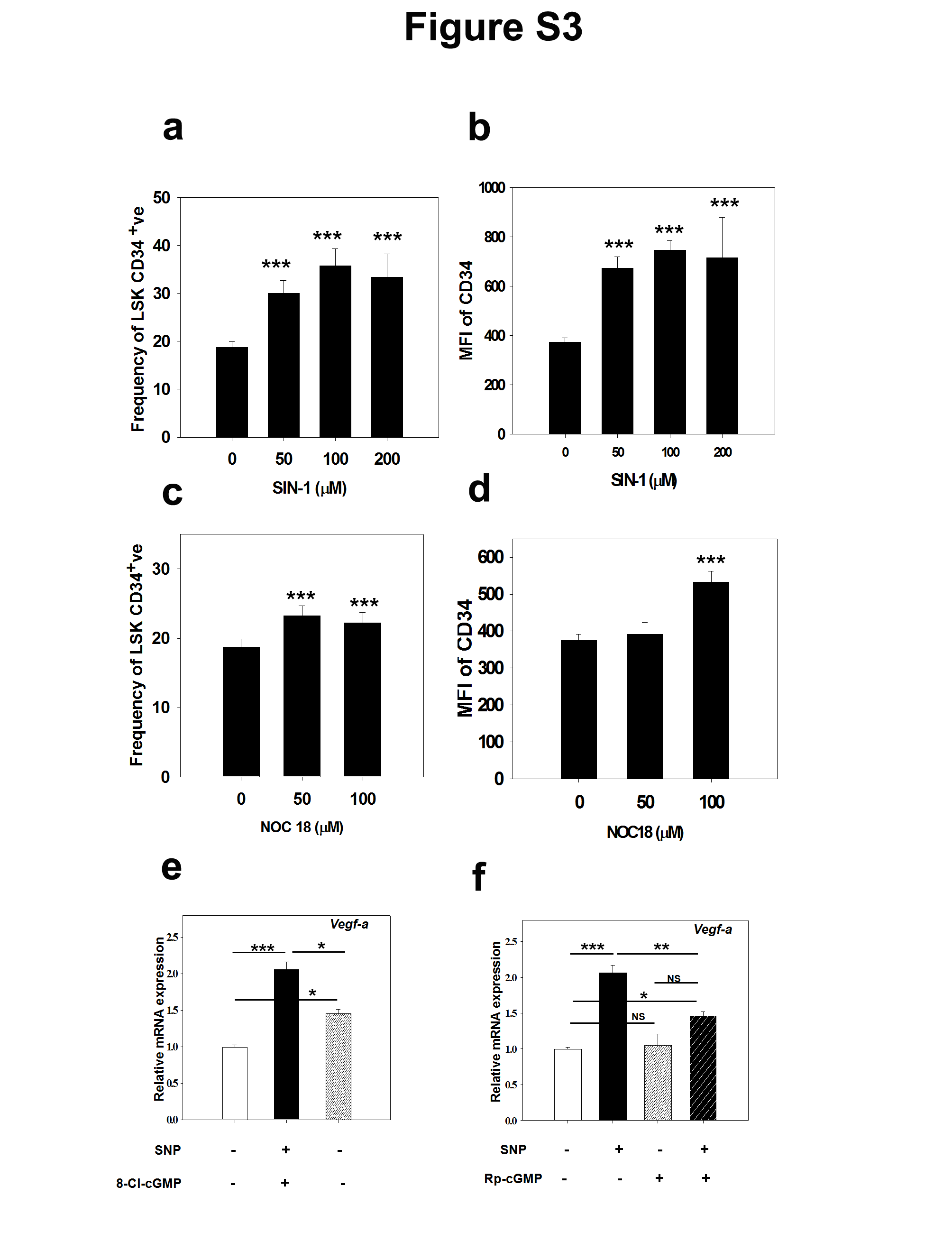

Supplement: Additional file 5: Figure S3. — Effects of NO donors on the membrane expression of CD34. Sort-purified LSK-CD34− cells were treated with various concentrations of SIN-1 and NOC-18 for 12 h. The increase in the frequency of LSK-CD34+ cells formed and MFI of CD34 fluorescence in the cells treated with SIN (a, b) and NOC-18 (c, d) are illustrated. ***p ≤ 0.001. Also see Fig. 3. (e, f) Expression of Vegf-a-specific mRNA in BMSCs directly treated with 100 μM 8-Cl-cGMP and BMSCs treated with 100 μM SNP in the presence of 100 μM Rp-cGMP is depicted. The data show that 8-Cl-cGMP significantly increased the Vegf-a expression in the BMSCs (e). Similarly, SNP-mediated increase in Vegf-a expression was significantly inhibited by Rp-cGMP (f). These data show that both chemicals exhibit desired activity. *p ≤ 0.05, **p ≤ 0.01, ***p ≤ 0.001. Also see Fig. 5. (TIF 512 kb) [file 13287_2016_433_MOESM5_ESM.tif]

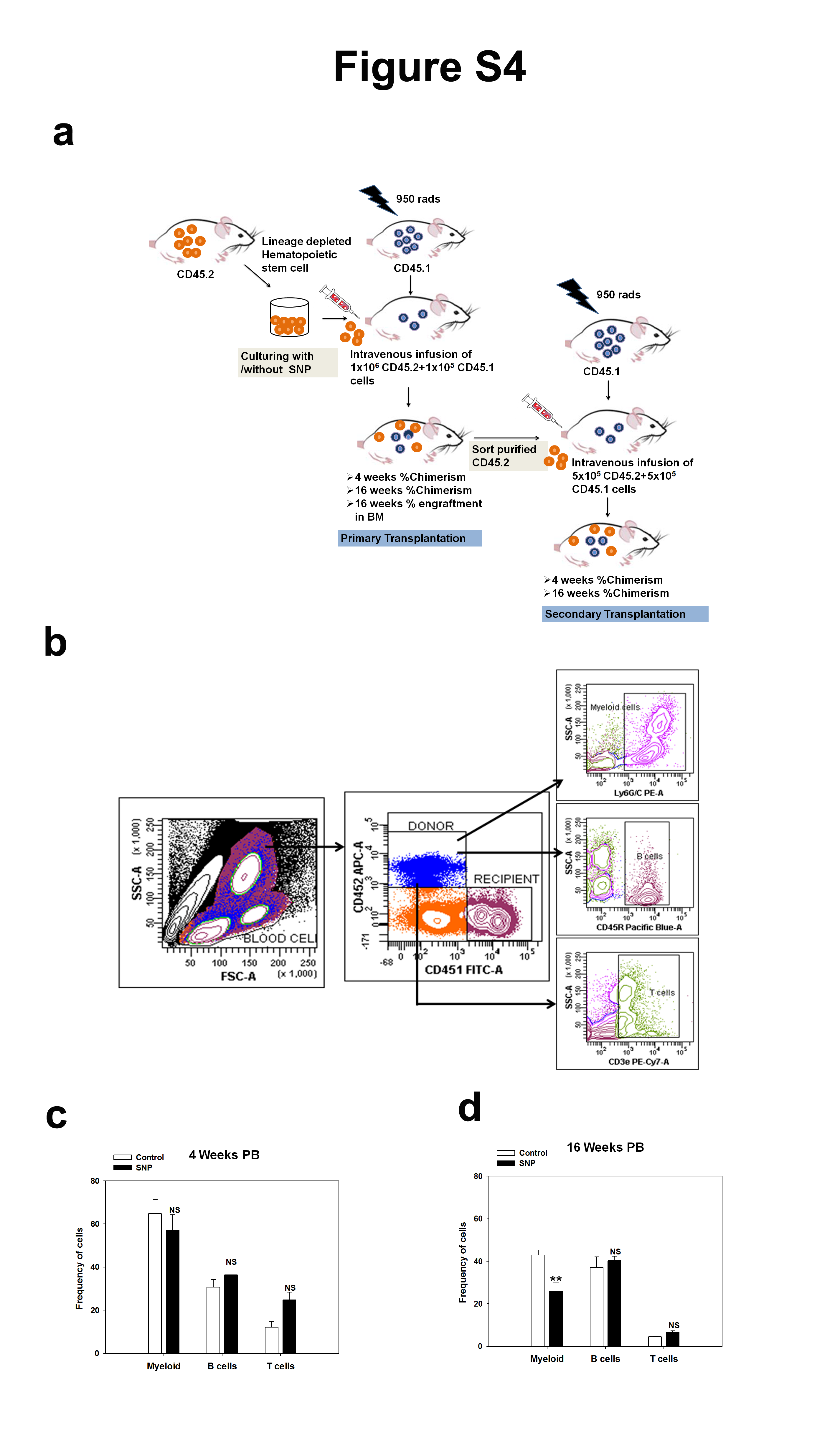

Supplement: Additional file 6: Figure S4. — Treatment of adult HSCs with NO donor does not induce lineage bias in regenerated blood cells. Lin− cells from adult mice (10–12 weeks; CD45.1)) were treated with 200 μM SNP for 3 days and were infused into irradiated recipients (8–10 weeks; CD45.2). Six mice were used per group. Donor cell chimerism was determined by flow cytometry of recipients’ PB. (a) Experimental scheme is illustrated. (b) Gating strategy used to analyze donor cell chimerism in the PB of recipient mice. (c, d) Lineage commitment in regenerated hematopoietic cells in the PB of recipients at 4 and 16 weeks post-transplant is illustrated. Also see Fig. 7. (TIF 2833 kb) [file 13287_2016_433_MOESM6_ESM.tif]

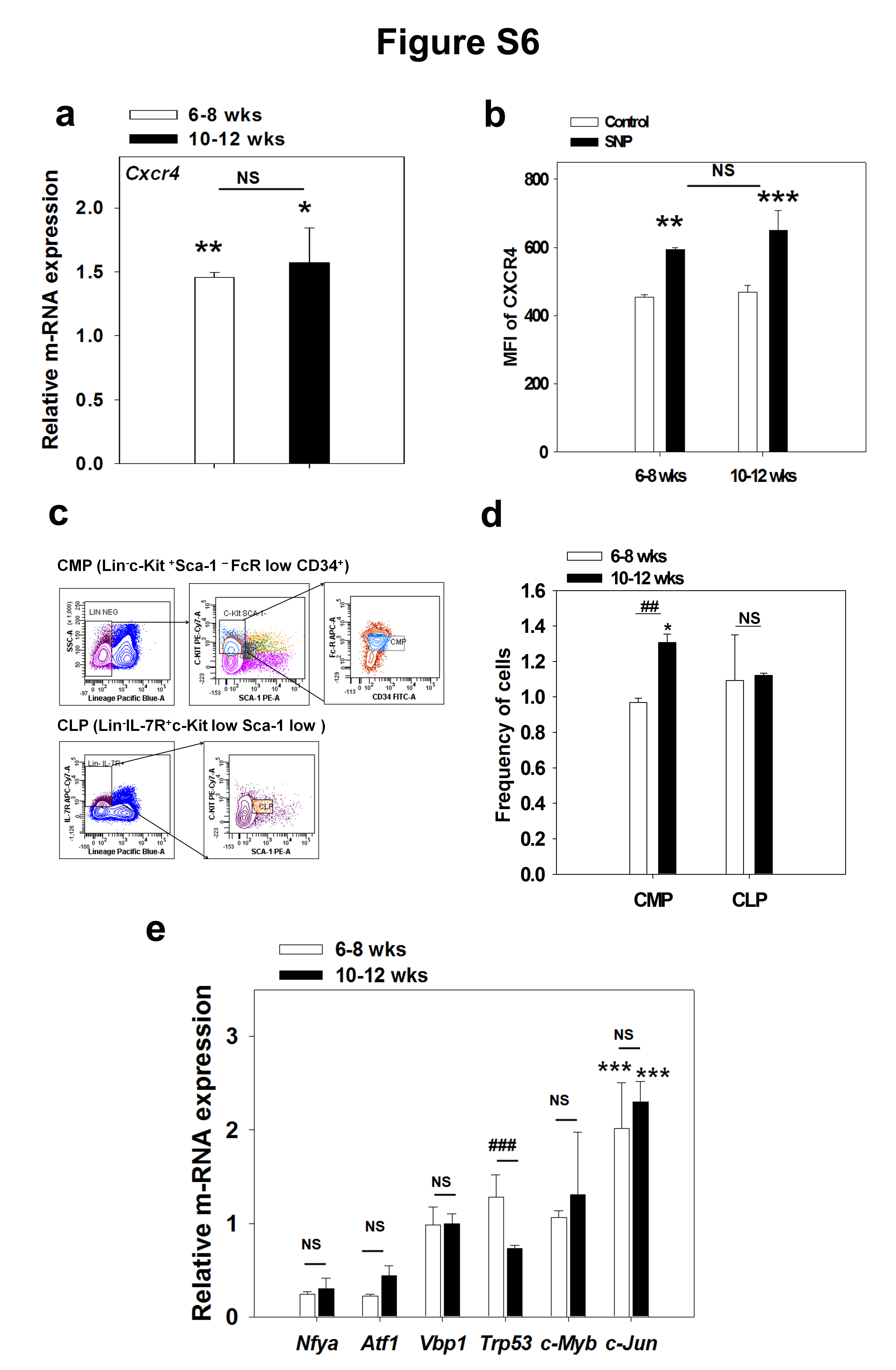

Supplement: Additional file 7: Figure S6. — NO upregulates Cxcr4 expression in murine HSCs. (a) Cxcr4-specific mRNA expression and (b) MFI of CXCR4 surface expression in SNP-treated juvenile and adult HSCs is graphically illustrated. NO increases myeloid commitment of adult HSCs. (c) Gating strategy applied for flow analysis of common myeloid progenitors (CMP) and common lymphoid progenitors (CLP) is illustrated. (d) Frequencies of CMP and CLP formed in SNP-treated adult and juvenile Lin− cells are graphically represented. Comparative transcription factor analyses of juvenile and adult HSCs treated with SNP. Sort-purified LSK-CD34− cells from juvenile mice (6–8 weeks) and adult mice (10–12 weeks) were treated with 200 μM SNP for 12 h and subjected to qRT-PCR analysis. (e) Comparative gene expression of various transcription factors in the treated cells is shown (N = 3). *p ≤ 0.05, **p ≤ 0.01, ***p ≤ 0.001, comparison between control and SNP-treated sets; ### p ≤0.001, comparison between SNP-treated juvenile versus SNP-treated adult HSCs. NS not significant. Also see Fig. 7. (TIF 2406 kb) [file 13287_2016_433_MOESM7_ESM.tif]

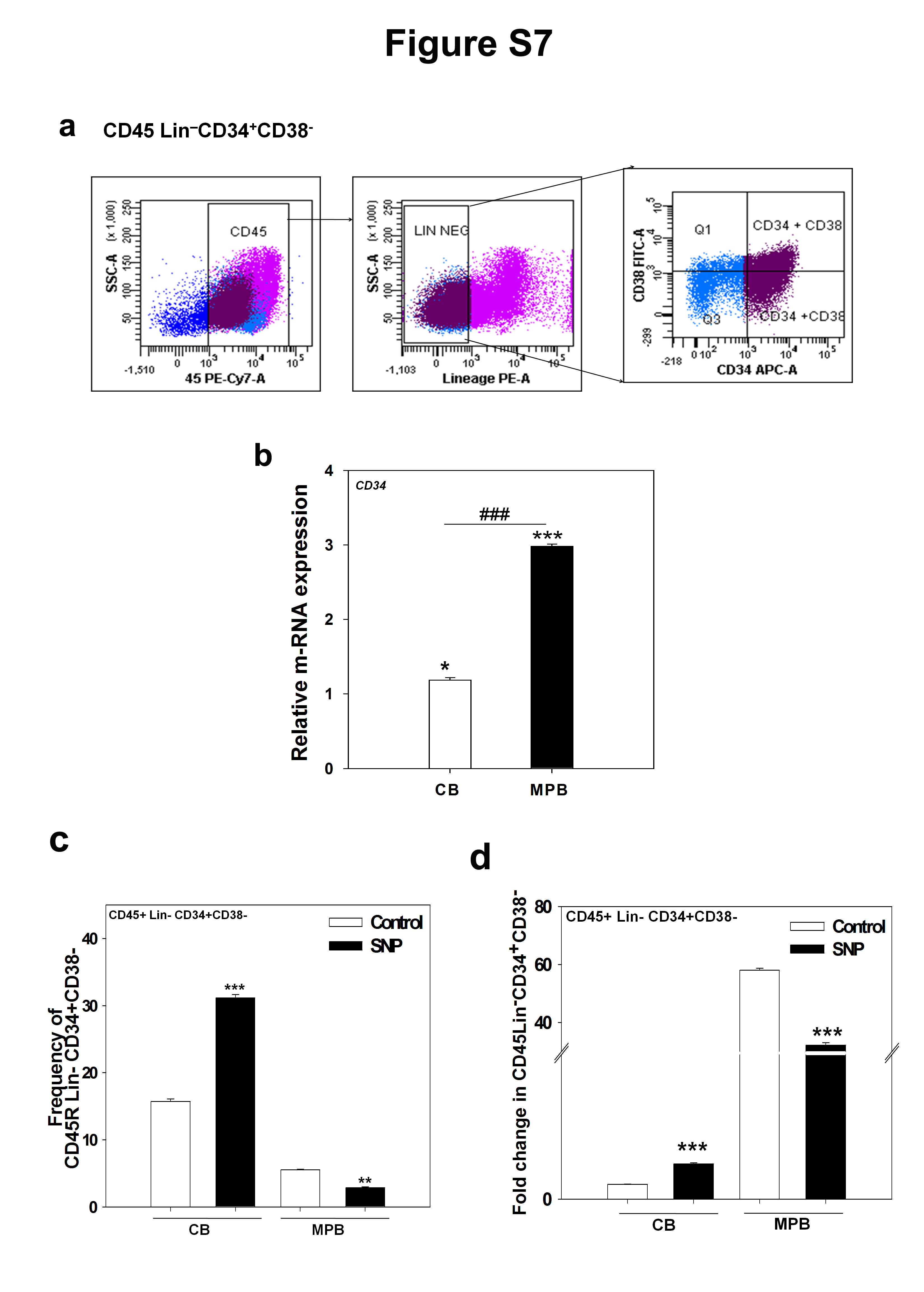

Supplement: Additional file 8: Figure S7. — NO exerts age-specific contrasting effects on human HSCs. (a) Gating strategy applied for human CD45+34+38− HSCs is illustrated. (b) SNP-mediated increase in CD34-specific mRNA and (c) frequency of CD34+38− HSCs in gated CD45+ population in SNP-treated CB and MBPL cells are graphically represented. (d) Fold-change in absolute numbers of CD45+34+38− HSCs generated in cultures over input CD45+34+38− HSC population is depicted. N = 3. *p ≤ 0.05, **p ≤ 0.01, ***p ≤ 0.001. (TIF 1949 kb) [file 13287_2016_433_MOESM8_ESM.tif]
